# Combinatorial functionalization with bisurea‐peptides and antifouling bisurea additives of a supramolecular elastomeric biomaterial

**DOI:** 10.1002/polb.24907

**Published:** 2019-12-03

**Authors:** Bastiaan D. Ippel, Boris Arts, Henk M. Keizer, Patricia Y. W. Dankers

**Affiliations:** ^1^ Institute for Complex Molecular Systems Eindhoven University of Technology PO Box 513 5600 Eindhoven Manitoba The Netherlands; ^2^ Department of Biomedical Engineering, Laboratory for Cell and Tissue Engineering Eindhoven University of Technology PO Box 513 5600 Eindhoven Manitoba The Netherlands; ^3^ Department of Biomedical Engineering, Laboratory of Chemical Biology Eindhoven University of Technology PO Box 513, 5600 Eindhoven Manitoba The Netherlands; ^4^ SyMO‐Chem B.V Den Dolech 2, 5612 Eindhoven Arizona The Netherlands

**Keywords:** additives, biointerfaces, peptides, screening, supramolecular biomaterials

## Abstract

The bioactive additive toolbox to functionalize supramolecular elastomeric materials expands rapidly. Here we have set an explorative step toward screening of complex combinatorial functionalization with antifouling and three peptide‐containing additives in a bisurea‐based supramolecular system. Thorough investigation of surface properties of thin films with contact angle measurements, X‐ray photoelectron spectroscopy and atomic force microscopy, was correlated to cell‐adhesion of endothelial and smooth muscle cells to apprehend their respective predictive values for functional biomaterial development. Peptides were presented at the surface alone, and in combinatorial functionalization with the oligo(ethylene glycol)‐based non‐cell adhesive additive. The bisurea‐RGD additive was cell‐adhesive in all conditions, whereas the endothelial cell‐specific bisurea‐REDV showed limited bioactive properties in all chemical nano‐environments. Also, aspecific functionality was observed for a bisurea‐SDF1α peptide. These results emphasize that special care should be taken in changing the chemical nano‐environment with peptide functionalization. © 2019 The Authors. *Journal of Polymer Science Part B: Polymer Physics* published by Wiley Periodicals, Inc. J. Polym. Sci., Part B: Polym. Phys. **2019**, 57, 1725–1735

## Graphical abstract

Adhesion of endothelial and smooth muscle cells on supramolecular biomaterials is regulated *via* combinatorial functionalization with bioactive‐peptide and antifouling additives. The modularity in this material system allows for fast preliminary screening of material properties and corresponding functioning of the incorporated additives. Changes in the chemical nano‐environment of bioactive peptides prove to be important factors in bioactive peptide functionality.

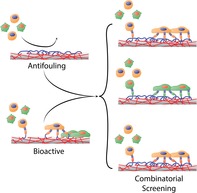

## INTRODUCTION

Biomaterial functionalization with various bioactive cues has been studied for several decades to be able to steer cellular behavior and positively influence the response upon implantation of the biomaterial.^1^ Inspiration for the design of these cues is often taken from natural extracellular matrix components.^2^ In direct approaches, extracellular matrix proteins have been used to coat or covalently functionalize biomaterials for improved bioactivity.^3^, ^4^ To induce or enhance adhesion of cells to synthetic materials, functionalization with short peptide mimics of adhesion sites in extracellular matrix proteins is a popular strategy. One of the most studied examples of such a peptide is the RGD sequence,^5^, ^6^, ^7^, ^8^, ^9^ found in for instance fibronectin and vitronectin. Besides peptides such as RGD, which induce cell adhesion in general, peptide sequences have been discovered for which specific cell types have an increased affinity over other cell types, due to increased expression of the binding motif on these cells.^2^ The REDV peptide, for which endothelial cell‐specificity has been reported,^10^, ^11^ attributed to binding to integrin α4β1,^11^, ^12^ has been extensively applied to enhance endothelialization of biomaterials.^13^, ^14^, ^15^, ^16^, ^17^, ^18^, ^19^, ^20^, ^21^, ^22^, ^23^, ^24^, ^25^, ^26^, ^27^, ^28^, ^29^, ^30^, ^31^, ^32^, ^33^, ^34^, ^35^, ^36^, ^37^, ^38^, ^39^ In another approach, a peptide derived from stromal cell derived factor‐1α (SDF1α), a chemoattractant of lymphocytes, monocytes, and progenitors cells, has been applied for improved *in situ* cellularization.^40^

Several parameters in the method of covalent tethering of peptides to a biomaterial surface influence the resulting functionality of the peptide. This includes, but is not limited to, the spacing between adhesive peptides,^41^ the length of the linker that is used to attach the peptide,^42^, ^43^, ^44^, ^45^, ^46^, ^47^ and the amino‐acids that flank the essential peptide sequence,^6^ which all suggest that tuning the chemical nano‐environment of peptides is of great importance for functionality of the peptides. However, a popular approach features the functionalization with both bioactive peptides, and antifouling moieties, to enhance specificity of the bioactivation and reduce adhesion of undesired entities to the functionalized biomaterial,^48^, ^49^, ^50^, ^51^ where the chemical environment of the peptides is designed to repel any aspecific interactions.

In our group, we apply a functionalization strategy based on specific supramolecular interactions,^52^, ^53^ that facilitate the non‐covalent incorporation of functional additives. In the past, adhesive properties of supramolecular biomaterials were improved through incorporation of additives, including additives with peptide functionality.^54^, ^55^, ^56^, ^57^ Here, the bisurea moiety is employed as the supramolecular motif, as an integral part of a segmented block‐copolymer as the hard‐segment. The mechanical properties of such hydrogen bonded elastomeric materials allow for application in biomedical applications ranging from heart valves^58^ and cardiac patches,^59^ to vascular grafts^60^, ^61^, ^62^ Bisurea‐based additives have been shown to self‐sort with matching motifs.^63^, ^64^, ^65^, ^66^ Furthermore, antifouling materials were formulated with oligo(ethylene glycol) (OEG)‐based bisurea additives, with a strong dependence of functionality on molecular design of the additive.^67^

**Figure 1:**
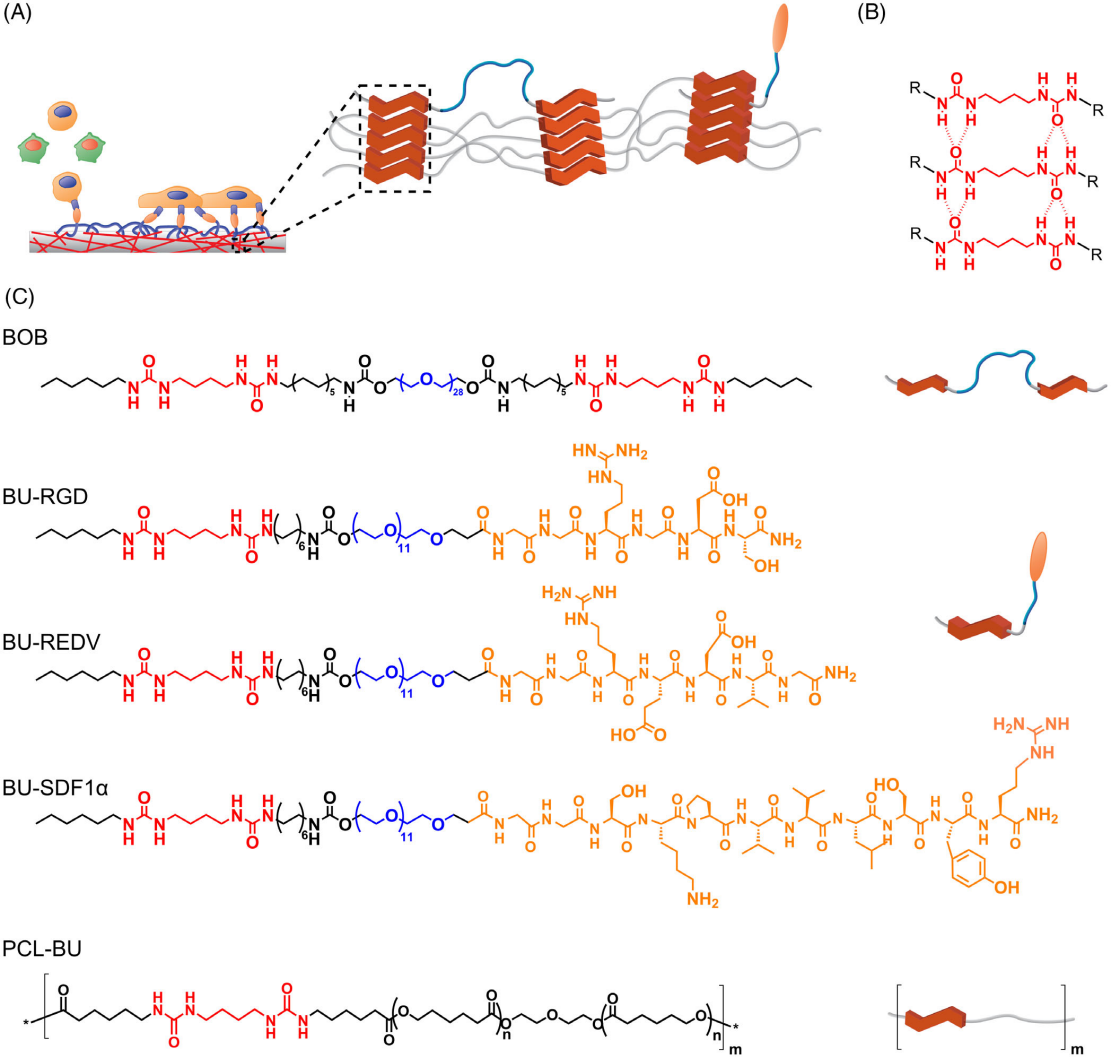
(A) Schematic representation of supramolecular approach for the combinatorial functionalization of BU‐peptides and BOB additives in the PCL‐BU base material and the resulting cell adhesion functionality. (B) Structural representation of the bifurcated hydrogen bonding pattern between bisurea moieties that facilitate the supramolecular assembly and incorporation of bisurea‐containing additives. (C) Structural representations of the PCL‐BU base material, BU‐OEG‐BU (BOB) antifouling additive, and the BU‐peptide conjugates (BU‐RGD, BU‐REDV, and BU‐SDF1α). [Color figure can be viewed at http://wileyonlinelibrary.com]

As a first step toward screening of bioactive supramolecular additives, using a combination of extensive surface characterization and functionality assessment, we functionalized the polycaprolactone‐bisurea (PCL‐BU) base material in combinatorial fashion with the best performing antifouling OEG‐based bisurea additive from our previous work, BU‐OEG‐BU (BOB),^67^ and three bioactive peptide additives. To this end three bisurea‐peptide conjugates, that is, BU‐RGD, BU‐REDV, and BU‐SDF1α (Fig. 1), were synthesized. Solution‐cast thin films of PCL‐BU, mixed with 2 and 4 mol% BOB, which is sufficient for non‐cell adhesive properties,^67^ and 1 and 4 mol% bisurea‐peptide additive, which has been shown to influence cellular adhesion,^57^ were prepared, and characterized with water contact angle measurements, atomic force microscopy, and X‐ray photoelectron spectroscopy. The cell‐adhesion of vascular endothelial and smooth muscle cells was studied to gain insight in the efficacy of the incorporated BU‐peptide additives, in co‐formulation with the antifouling BOB. Moreover, this combinatorial approach allowed us to systematically study the functionality of the mixtures with bisurea‐peptides in different supramolecular nano‐environments. These results can determine the predictive value of extensive surface characterization, in view of further screening of new bioactive supramolecular additives.

## EXPERIMENTAL

### Materials and Methods

All reagents, chemicals, materials, and solvents were obtained from commercial sources and were used as received. All solvents were of AR quality. Moisture or oxygen‐sensitive reactions were performed under an argon atmosphere. In the synthetic procedures, equivalents (eq) are molar equivalents. Analytical thin layer chromatography was performed on Kieselgel F‐254 precoated silica plates. Silica column chromatography was carried out on Screening Devices B.V. silica gel (flash: 40–63 μm mesh or normal: 60–200 μm mesh). ^1^H‐NMR, ^13^C‐NMR, and ^19^F‐NMR spectra were recorded on a Bruker Avance III HD spectrometer at 298 K. Chemical shifts are reported in ppm downfield from TMS at room temperature. Abbreviations used for splitting patterns are s = singlet, d = doublet, t = triplet, q = quartet, qn = quintet, m = multiplet, and br = broad. IR spectra were recorded on a Shimadzu IR Affinity‐1 or a Perkin Elmer Spectrum Two ATR FT‐IR machine. Matrix‐assisted laser desorption ionization time‐of‐flight mass spectrometry (MALDI‐TOF MS) was measured on a Bruker Autoflex Speed using a 2‐((2E)‐3‐(4‐t‐butylphenyl)‐2‐methylprop‐2‐enylidene)malononitrile (DCTB) matrix. HPLC‐PDA/ESI‐MS analyses were performed using a Shimadzu LC‐10 AD VP series HPLC coupled to a diode array detector (Finnigan Surveyor PDA Plus detector, Thermo Electron Corporation) and an ion‐trap (LCQ Fleet, Thermo Scientific), applying a 95/5 to 5/95 water/acetonitrile gradient, with this eluent always containing 0.1 v/v% formic acid.

### Synthesis

PCL‐BU^61^, ^64^ and BU‐OEG‐BU^67^ were synthesized as described previously. The synthesis of the bisurea‐carboxylic acid (BU‐COOH) and bisurea‐TFP‐ester precursor and additional analyses of intermediate products are described in the supporting information.

### Bisurea‐SDF‐1α

Protected SDF‐1α peptide (Supporting Information Scheme S2, **5**) was synthesized on solid‐phase using Sieber resin (1.04 g, loading 0.61 mmol/g). Amino acid elongation involved treating the resin with 20% piperidine in NMP to cleave the Fmoc‐protecting group, followed by coupling of the amino acid (1.34 mmol/g resin) using N,N‐diisopropylethylamine (DIPEA, 2 eq) as base and *O*‐benzotriazole‐*N*,*N*,*N'*,*N′*‐tetramethyluronium hexafluorophosphate (HBTU, 1 eq) as activator. To cleave the protected peptide from the resin, a 1% trifluoroacetic acid (TFA) in dichloromethane (DCM) solution was added to the resin and after 3 min the solution was removed and the resin was rinsed with DCM (3x). This procedure was repeated 10 times, and the collected organic layers were combined and washed with water. The DCM was removed *in vacuo*. The residue was the crude product that was purified by normal phase silica chromatography (start MeOH/CHCl_3_ 5/95, stepwise increase of MeOH fraction to 7%, to 10%, to 15% to 20%) to afford 0.25 g (49%) of *protected*‐SDF‐1α product as a white powder. HPLC‐MS(ESI) R_t_ = 5.95 min m/z calcd (C_82_H_136_N_16_O_19_S) 1682.1; found 842.7 [M + 2H]^2+^, 1682.8 [M + H]^+^.

Bisurea‐TFP‐ester (Supporting Information Scheme S1, **4**) (60.0 mg, 0.049 mmol) was dissolved in DMF (5 mL) with some heating. *Protected*‐SDF‐1α **5** (106.3 mg, 0.063 mmol) and DIPEA (0.085 mL, 0.49 mmol) were added, and the mixture was stirred for 45 min at 45°C and overnight at room temperature under an inert argon atmosphere. The solvent was removed *in vacuo*, and the residue was co‐evaporated twice with toluene. Elution over normal phase silica (start MeOH/CHCl_3_ 5/95, stepwise increase of MeOH to 10%, to 15%) afforded 115 mg (85%) of the C6‐U4U‐C12‐dPEG12‐C2‐*protected*‐SDF‐1α product (Supporting Information Scheme S2, **6**). HPLC‐MS(ESI) R_t_ = 8.68 min m/z calcd (C_134_H_237_N_21_O_36_S) 2750.5; found 688.3 [M + 4H]^4+^, 917.4 [M + 3H]^3+^, 1375.7 [M + 2H]^2+^.

To C6‐U4U‐C12‐dPEG12‐C2‐*protected*‐SDF‐1a **6** (115 mg, 0.042 mmol), a mixture of TFA/triisopropylsilane (TIS)/H_2_O (95/2.5/2.5) was added. The resulting solution was stirred for 1 h under an argon atmosphere. The solvent was removed *in vacuo*, and the residue was coevaporated twice with toluene. Ether (10 mL) was added to the residue, the suspension was stirred, and the superfluent was decanted off (2x). Acetonitrile (5 mL) was added to the solid, and after stirring of the mixture, ether (15 mL) was added, followed by centrifugation of the suspension to collect the solid. Finally, the solid was dissolved in water/MeCN (90/10) and was freeze‐dried to give Bisurea‐SDF1α product (Supporting Information Scheme S2, **7**) (92 mg, 98%) as a white powder. HPLC‐MS(ESI) R_t_ = 4.93 min m/z calcd (C_104_H_189_N_21_O_31_) 2229.8; found 558.3 [M + 4H]^4+^, 744.0 [M + 3H]^3+^, 1115.4 [M + 2H]^2+^.

### Bisurea‐REDV

GGREDVG was manually synthesized through Fmoc‐based solide phase peptide synthesis. The peptide was synthesized on a Rink‐amide resin (0.61 mmol/g, 50 μmol scale) in dimethylformamide (DMF) as a solvent with O‐(1H‐6‐Chlorobenzotriazole‐1‐yl)‐1,1,3,3‐tetramethyluronium hexafluorophosphate (HCTU) as an activator and DIPEA as a base (4:4:16 equivalent of amino acid/HCTU/DIPEA to the resin). All amino acids were coupled in duplicates. Fmoc deprotection was achieved with 20 (v/v)% piperidine in DMF. Bisurea‐carboxylic acid was coupled on the solid support to the N‐terminus of the peptide with a ratio of 2:1.2:5 equivalent of BU‐COOH/HATU/DIPEA to the resin. After overnight reaction, bisurea‐peptide was cleaved using a mixture of TFA, TIS, and mQ (95/2.5/2.5 (v/v)%). The BU‐peptide conjugate was obtained pure directly after synthesis indicated by HPLC‐MS(ESI). TFA ions were exchanged by chloride ions *via* two cycles of dissolving the peptide in 2 mM HCl and freeze drying. Lyophilisation yielded a white powder. HPLC‐MS(ESI) R_t_ = 5.31 min m/z calcd (C_78_H_146_N_16_O_28_) 1755.1 g mol^−1^; found [M + 1H]^1+^ = 1756.20 g mol^−1^, [M + 2H]^2+^ = 879.00 g mol^−1^, [M + 3H]^3+^ = 586.42 g mol^−1^.

### Preparation of Polymer Films

PCL‐BU and additives were dissolved in 1,1,1,3,3,3‐hexafluoroisopropanol (HFIP, Fluorochem) at a concentration of 7.4 mM, where the molar mass of a single repeating unit was used for PCL‐BU, which is depicted in Figure 1. The solutions were mixed in appropriate molar ratios through the combination of volumes in the same respective ratios. This resulted in solutions of approximately 20 mg mL^−1^, of which 25 or 50 μL was cast on 10 or 14 mm glass coverslips, respectively. The films were dried to air for several hours, before final drying in vacuum overnight.

### Atomic Force Microscopy

Atomic Force Microscopy was performed on a Digital Instruments Multimode Nanoscope IIIa, and a Digital Instruments Dimension 3100 Nanoscope IIIa. Phase and height images of solution‐cast films were recorded in the tapping mode regime in air at room temperature using silicon cantilever tips (PPP‐NCHR). Images were processed using Gwyddion software (version 2.43), and the root mean square roughness was extracted from 1 × 1 μm height images.

### X‐Ray Photoelectron Spectroscopy

XPS was performed using a Thermo Scientific K‐Alpha spectrometer equipped with a monochromatic, small‐spot X‐ray source, and a 180° double focusing hemispherical analyzer with a 128‐channel detector. Coverslips were secured using double‐sided carbon tape, and spectra were obtained using an aluminum anode (Al Kα, 1486.7 eV, 72 W). Survey scans were measured at a pass energy of 200 eV and region scans at a pass energy of 50 eV. Scans were analyzed using CasaXPS software (version 2.3.18). For quantification, the high resolution scans of carbon, oxygen, and nitrogen were used.

### Water Contact Angle Measurements

An OCA30 machine (DataPhysics), operated with SCA20 software (version 4.1.13), was used to determine static water contact angles on solution‐cast films at room temperature. Five microliters of Milli‐Q water droplets were applied to the surface through a needle, and the contact angle was measured at the polymer‐air‐water interface 5 s after deposition of the droplet.

### Endothelial Cell Culture

Both Human Umbilical Vein Endothelial Cells (HUVEC, Lonza) and human Aortic Endothelial Cells (hAECs, Lonza) were cultured in endothelial growth medium (Lonza and PromoCell, endothelial basal medium supplemented with endothelial cell growth factors, and additional Pen/Strep for the supplemented PromoCell medium). Culture flasks were coated with 0.1% gelatin in PBS for approximately 15 min at 37 °C prior to seeding the cells, which were passaged before reaching 80% confluency. Cells were harvested using trypsin/EDTA and used for experiments up to passage 6.

### Smooth Muscle Cell Culture

Human aortic smooth muscle cells (hASMCs, Lonza) were cultured in smooth muscle growth medium, consisting of medium 231 supplemented with smooth muscle cell growth factors (and additional Pen/Strep). Cells were harvested using trypsin/EDTA and used for experiments up to passage 5.

### Cell Adhesion Assay

Solution‐cast films on 14‐mm glass coverslips were secured in an adapted Transwell as described before^67^, ^68^ and sterilized under UV for 15 min. Endothelial cells were seeded at a density of 40.000 cells cm^−2^ and smooth muscle cells at 25.000 cm^−2^. Five hundred microliters of cell‐suspension was used inside the inserts, and 1 mL of appropriate growth medium was added outside the insert after establishing the inserts did not leak immediately. Cells were cultured for 24 h at 37 °C and 5% CO_2_, after which non‐adherent cells were aspirated and the surfaces were washed with PBS with subsequent fixation in 3.7% formaldehyde. The actin‐cytoskeleton was stained with ATTO488 conjugated phalloidin, and nuclei were counterstained with 4′,6‐diamidino‐2‐phenylindole (DAPI). Samples were visualized using a Leica DMi8s microscope. Furthermore, the surface covered by cells was determined by binarizing fluorescence micrographs of the phalloidin‐stained samples into background and cells in ImageJ (NIH, version 1.48). The percentage of foreground pixels was used as a measure for the area occupied by cells. Three images taken with a 10x objective were measured per condition.

### Endothelial Cell and Smooth Muscle Cell Co‐Culture

HAECs and hASMCs were expanded as before. Prior to harvesting, medium was aspirated from the cells, and cells were incubated for 30 min with 10 μM CellTracker Green CMFDA and Orange CMTMR (Invitrogen), respectively, for 30 min. The cells were then washed with PBS twice and harvested. Both HAECs and HASMCs were resuspended in smooth muscle cell growth medium and combined 1:1 for a total of 80.000 cells mL^−1^. Five hundred microliters of mixed‐cell suspension was seeded on each of the coverslip, which corresponds to approximately 20.000 hAECs and 20.000 hASMCS cm^−2^. After 24 h of culture, the cells were visualized before and after fixation in 3.7% formaldehyde and counterstaining with DAPI using a Leica DMI8s microscope.

## RESULTS AND DISCUSSION

### Material Toolbox

A materials toolbox was designed for the first step toward screening complex combinatorially functionalized supramolecular biomaterials, where we aimed to use structure–property relations to predict functionality for these mixtures. The bisurea‐peptide conjugates were successfully synthesized with fmoc solid phase peptide synthesis and subsequent coupling of a bisurea‐OEG‐COOH to the N‐terminus on the resin. The ethylene glycol linker should ensure proper exposure at the surface of the mixtures and is hypothesized to facilitate better mixing in the combination with the OEG‐based antifouling BOB,^69^ of which the design was described previously.^67^

### Surface Characterization

**Figure 2:**
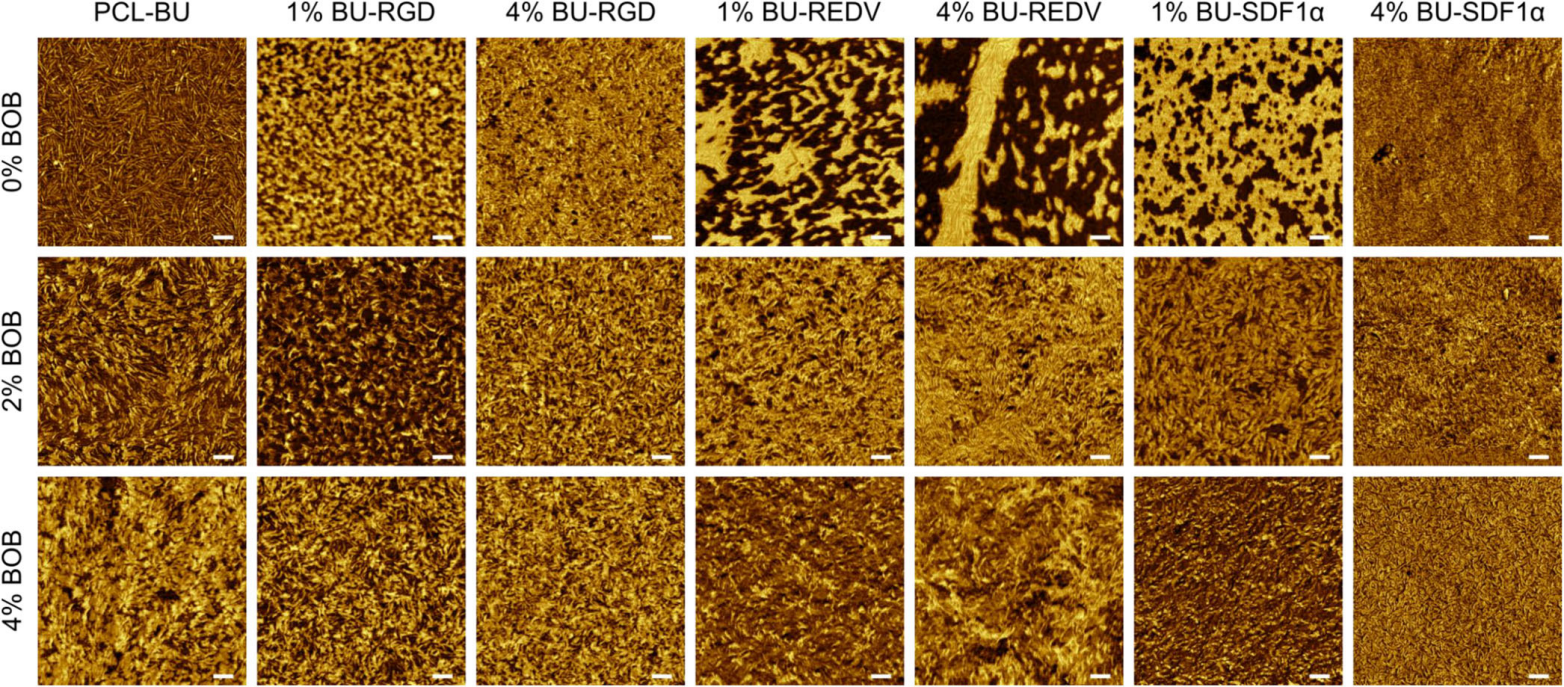
Atomic force microscopy phase images recorded in tapping mode in air of solution‐cast films of PCL‐BU with mixtures of BU‐OEG‐BU and the BU‐peptide conjugates. Scale bars represent 100 nm. [Color figure can be viewed at http://wileyonlinelibrary.com]

Atomic force microscopic analysis allows for the surface morphology of the solution‐cast films to be probed, which can give insight in the influence of additives on the combinatorial self‐assembly with the supramolecular base material and the other additives. In phase images of the pristine PCL‐BU, a characteristic fibrous morphology is visible (Fig. 2),^63^, ^65^, ^66^, ^70^ where the brighter domains are attributed to the hard‐phase made up of self‐assembled bisurea fibers. Upon incorporation of the non‐cell adhesive BOB additive, a fibrous morphology was retained, which indicates proper incorporation of the additive in the base material. The increased presence of brighter domains such as with 2 and 4% BOB are associated with presence of poly(ethylene glycol) at the surface, similar to previously reported results.^67^ When the bisurea‐peptide additives were mixed with the PCL‐BU polymer, brighter domains were again observed, but here in a different capacity. For the BU‐RGD and BU‐SDF1α, small fibrillar structures can be resolved within the brighter domains. The BU‐REDV formed larger domains, in which these fibrous structures are clearly present. In the background darker areas, the fibrous structure that resembles the pristine PCL‐BU can still be detected, which is especially apparent in the surfaces with only BU‐REDV. Such a fibrous morphology is not self‐evident upon incorporation of a supramolecular peptide additive.^71^ These surface morphology changes upon formulation of various additives observations indicate that changes in additive design, and therefore molecular interactions with the PCL‐BU base material, influence the assembly of base material and additive. For all the surfaces with combinations of the BU‐peptides and the BOB additive in the PCL‐BU base material, the surface appeared to be saturated with additive. The fibrous morphology that was observed for the bisurea‐peptides separately was retained upon combination with BOB, except for the combination of 4% BOB and 1% BU‐SDF1α, where the surface was more of an amorphous nature. However, the separated domains of peptide, that were present most obvious with BU‐REDV and to a lesser extent with BU‐SDF1α and BU‐RGD, are no longer discernible upon combination with BOB and the distribution of the additives over the surfaces is more homogeneous. In the height images, the same trend is observed (Supporting Information Fig. S1) and the nanoscale roughness of these surfaces is highest for the surfaces with only BOB, and smaller than 4 nm for all films with BU‐peptide additives (Supporting Information Table S1). These observations indicate a retained presence of the peptides at the surface, in the case of co‐formulation with the BOB additive.

**Figure 3:**
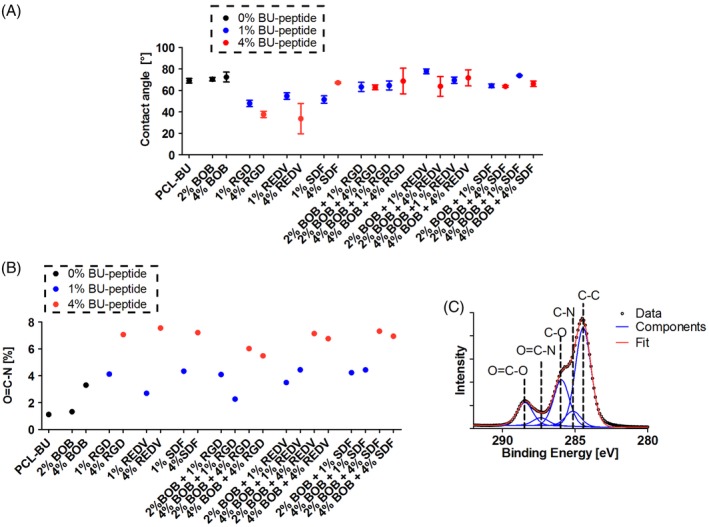
(A) Water contact angles measured on solution‐cast films of PCL‐BU with mixtures of BU‐OEG‐BU and the BU‐peptide conjugates. Data are represented as mean ± SD. (B) Quantification of fraction of O=C—N carbon species from narrow scan XPS carbon spectra. (C) Example of fit in narrow scan XPS carbon spectra for PCL‐BU + 1% BU‐RGD. The data are represented by the black circles, the individual modeled components by blue curves, and the sum of the components, or the total fit by the red curve. [Color figure can be viewed at http://wileyonlinelibrary.com]

Water contact angle measurements give an indication for the macroscale hydrophilic properties of an interface. In general, functionalization with hydrophilic moieties will result in an increased hydrophilicity of the surface.^72^ However, when the BOB additive was incorporated in PCL‐BU previously, no increase in hydrophilicity was observed similar to what was observed here (Fig. 3).^67^ The inclusion of the different BU‐peptide conjugates in the PCL‐BU base material led to different changes in surface hydrophilicity. Incorporation of 1 and 4% BU‐RGD results in a stark decrease in contact angle. A similar concentration dependent decrease in contact angle resulted from the incorporation of BU‐REDV, which is in line with previous efforts with REDV surface functionalization.^15^, ^28^, ^36^, ^73^ Interestingly, the incorporation of 1% BU‐SDF1α results in a lower contact angle compared to 4% BU‐SDF1α, which is comparable to that of pristine PCL‐BU. In combination with the noncell adhesive BOB additive, the incorporation of BU‐peptide conjugates resulted in contact angles that did not deviate considerably from pristine PCL‐BU, and no concentration dependence can be discerned.

Using X‐ray photoelectron spectroscopy, the chemical composition of the surfaces with these self‐assembled complex mixtures was investigated. In general, an increase in the contribution of nitrogen was detected upon incorporation of the additives, which can be explained by the presence of additional urea, urethane, and amide bonds in both the BOB and BU‐peptide additives (Supporting Information Table S2). More direct evidence for the presence of the peptides at the surface arose from deconvolution of the C 1s narrow scans of these surfaces, where the contribution of different carbon species was fitted (Fig. 3C). Evidently, the fraction of carbon species that could be attributed to O=C—N bonds, which are abundant in the backbone of peptides, can be correlated to the extent of the incorporation of the BU‐peptide additives (Fig. 3, Supporting Information Table S3, additional XPS spectra can be found in the Supporting Information), which further substantiates the determined peptide presence at the surface of these films. The C 1s component attributed to C—O that is distinctive of the ethylene glycol in both the BOB additive and the OEG linker present in the bisurea‐peptide additives, increases clearly with BOB concentration but has a poor positive correlation to amount of oligo(ethylene glycol) (Supporting Information Fig. S9). The chemical and morphological characterization of these complex material interfaces suggests a persistent exposure of the peptides at the surface, which was not negated by combinatorial self‐assembly with the antifouling BOB additive. The analysis of macro‐scale hydrophilic properties however cannot distinguish for surfaces with or without BU‐peptides.

### Screening of Cell‐Adhesive Properties of Complex Co‐Formulations for Endothelial and Smooth Muscle Cells

**Figure 4:**
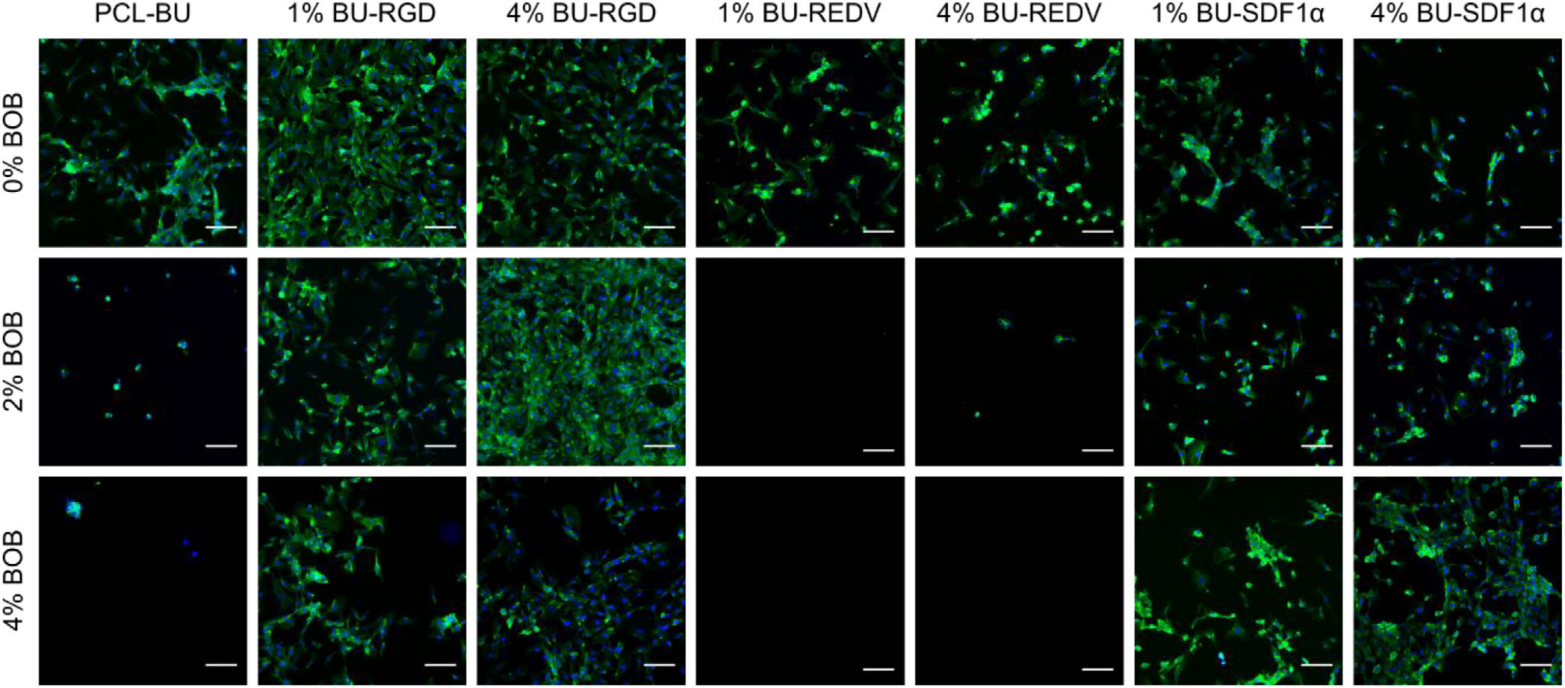
Fluorescence micrographs of human umbilical vein endothelial cells cultured for 24 h on solution‐cast films of PCL‐BU functionalized with mixtures of anti‐fouling BOB, and BU‐peptide conjugates. The actin cytoskeleton is represented in green, nuclei in blue. Scale bars indicate micrometer. [Color figure can be viewed at http://wileyonlinelibrary.com]

Human umbilical vein endothelial cells (HUVEC) and human aortic smooth muscle cells (hASMC) were cultured on the solution‐cast films as a measure for the effectivity and bioactive properties of these co‐assemblies of bisurea‐peptide additives and antifouling BOB. On PCL‐BU, the HUVECs adhere and spread and show the tendency to form small colonies (Fig. 4). Upon functionalization with BOB, the adhesion and spreading of HUVECs was significantly decreased (Supporting Information Fig. S10), and only small clusters of rounded cells were present, similar to results on the same mixtures with a myofibroblast‐like cells.^67^ Incorporation of the generic cell‐adhesive BU‐RGD increased the adhesion and spreading of the HUVECs and more actin stress‐fibers can be seen (Fig. 4). Supramolecular incorporation of BU‐REDV only did not improve adhesion of the HUVECs. Functionalization with 1% BU‐SDF1α resulted in similar HUVEC adhesion and spreading compared to pristine PCL‐BU, whereas the adhesion on surfaces with 4% BU‐SDF1α may even be slightly decreased. The adhesion of the endothelial cells to the surfaces with the mixtures of BOB and BU‐SDF1α might be explained by the presence of the CXCR4 receptor on the endothelial cell membrane, even though this receptor has no obvious adhesive function for the endothelial cells.^74^ Strikingly, the integrin α4β1 that has been shown to be present in endothelial cells^11^ is not able to induce cell adhesion through binding with the REDV on the passivated BOB‐functionalized surfaces. An increased concentration of 6 and 8 mol% BU‐REDV, both alone and in combination with the non‐cell adhesive BOB, also did not result in an increased adhesion for the endothelial cells (Supporting Information Fig. S11).

**Figure 5:**
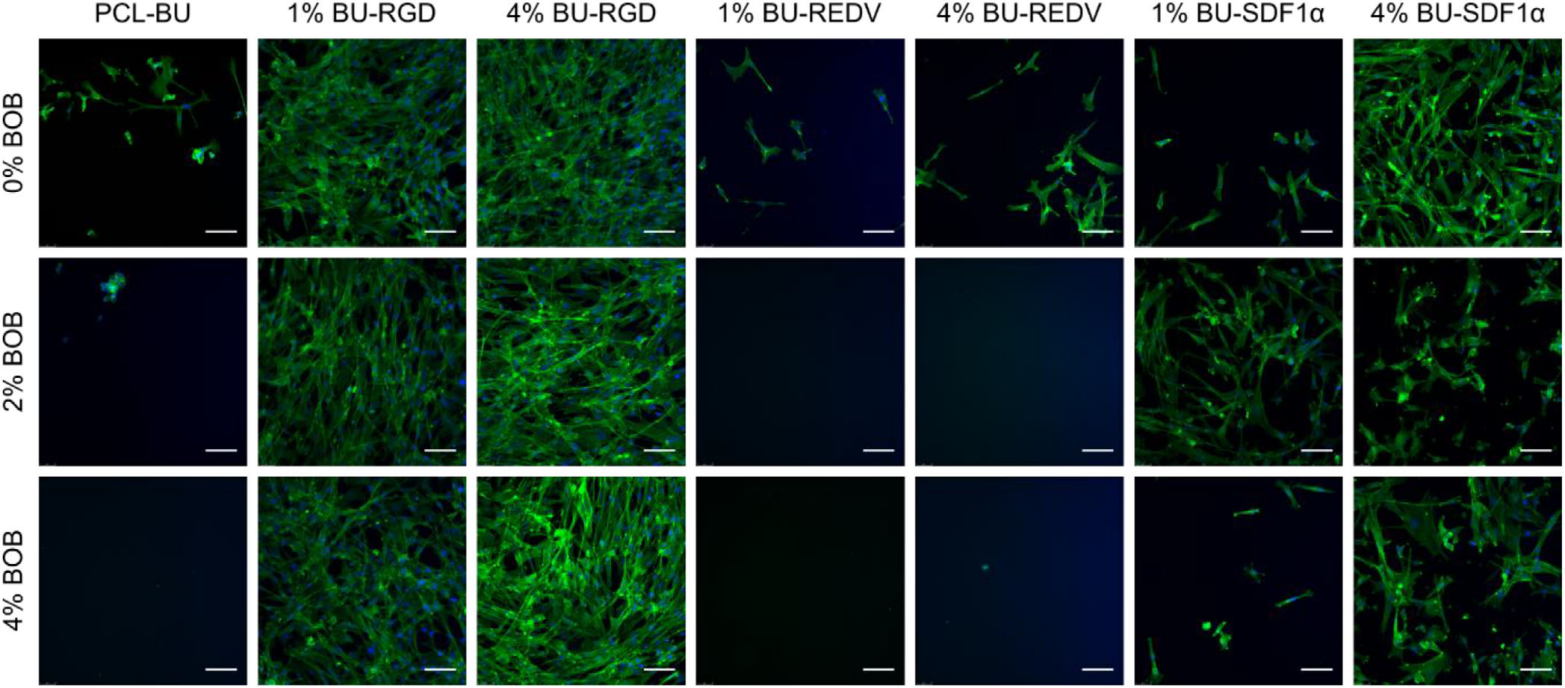
Fluorescence micrographs of human aortic smooth muscle cells cultured for 24 h on solution‐cast films of PCL‐BU functionalized with mixtures of anti‐fouling BU‐OEG‐BU and BU‐peptide conjugates. The actin cytoskeleton is represented in green, nuclei in blue. Scale bars indicate 100 μm. [Color figure can be viewed at http://wileyonlinelibrary.com]

For human Aortic Smooth Muscle cells, this panel of material mixtures showed similar cell‐adhesive properties, even though the adhesion on pristine PCL‐BU was not particularly good. Cell spreading and adhesion was significantly decreased through incorporation of the non‐cell adhesive BOB additive. The adhesive properties were efficiently restored in the combinatorial functionalization with BU‐RGD, which resulted in clearly spread cells that depicted a defined actin cytoskeleton (Fig. 5) Here, expectedly the functionalization with BU‐REDV did not increase smooth muscle cell adhesion compared to pristine, and neither did the combination of BOB and BU‐REDV result in adhesion of SMCs (Supporting Information Fig. S10). However, comparable to the endothelial cells, both incorporation of BU‐SDF1α alone and the composite of BU‐SDF1α and BOB resulted in the adhesion of SMCs. Yet the morphology of these cells was less spread compared to BU‐RGD functionalized surfaces, and the actin cytoskeleton was less defined, which may indicate less tight attachment to the biomaterial surface. Interestingly, the amount of cells that adhered on the materials containing both BOB and BU‐SDF1α showed a negative correlation to the BU‐SDF1α concentration for 2% BOB, where less cells adhered on the surface with 4% BU‐SDF1α compared to 1% BU‐SDF1α, whereas this correlation was positive for the combination with 4% BOB, where more cells adhered with 4% BU‐SDF1α compared to 1% BU‐SDF1α. This observation cannot be correlated to neither the macro‐scale hydrophilicity, which is comparable for all four combinations and close to that of pristine PCL‐BU, nor the amount of peptide, if the O=C—N component is taken as a measure.

**Figure 6:**
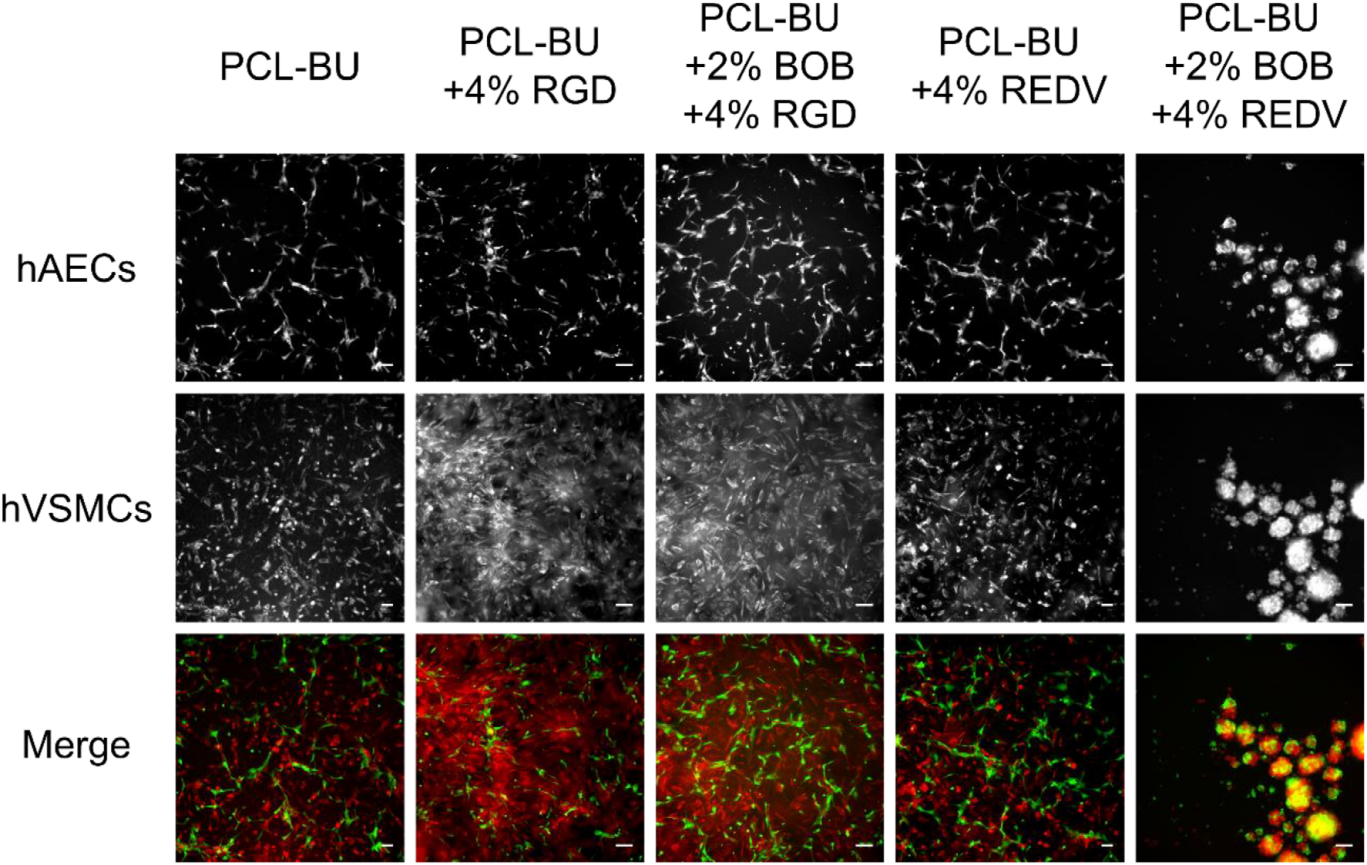
Fluorescence micrographs of endothelial cells and smooth muscle cells co‐cultured on thin films op PCL‐BU with of PCL‐BU functionalized with mixtures of anti‐fouling BU‐OEG‐BU and BU‐peptide conjugates. Endothelial cells (top row) are stained in green, and smooth muscle cells (middle row) are stained in red. Scale bars represent 100 μm. [Color figure can be viewed at http://wileyonlinelibrary.com]

In the single cell‐type cultures, comparable adhesive behavior was observed. Since literature states the ability of the REDV peptide to provide specificity in adhesion for endothelial cells over smooth muscle cells, a co‐culture experiment was conducted. For this human Aortic Endothelial Cells were used, which showed a similar response in single culture to REDV functionalized surfaces (Supporting Information Fig. S5) and the human aortic smooth muscle cells for a more fair comparison. For pristine PCL‐BU, less ECs adhere compared to the SMCs (Fig. 6), which is not altered by incorporation of 4% BU‐RGD, 4% BU‐REDV, or the combination of 2% BOB and 4% BU‐RGD. However, the adhesion of SMCs seemed to be increased for BU‐RGD functionalized films. Congruent with the results for the single‐cell cultures, the co‐formulation of BU‐REDV with BOB was not able to negate the non‐cell adhesive functionality of BOB for both ECs and SMCs in co‐culture.

### General Discussion

The modular supramolecular approach allowed for this study on the functionality and presentation of BU‐peptide conjugates, both alone and in combination with the non‐cell adhesive BOB additive, in varying ratios. The combination of BU‐RGD and antifouling BOB in the PCL‐BU base polymer resulted in surface where RGD presentation restored biofunctional properties. Cells adhered readily, despite the OEG of the BOB being present at the surface. Even though obvious differences between the co‐assemblies of the BU‐peptides and the BOB did not emerge from screening of their surface properties, the BU‐REDV and BU‐SDF1α functionalized surfaces have unequivocally different functionality in terms of cellular adhesion. Many reports in the literature describe the functionalization of biomaterials with RGD peptides for increased bioactivity through improved adhesion of numerous cell types.

Numerous studies describe the functionalization of biomaterials with the RGD peptide for increased bioactivity through improved adhesion of numerous cell types,^6^, ^9^ including on materials where antifouling linkers or substrates were applied as the base.^75^ Sufficient anchoring of RGD‐moieties to biomaterials is a prerequisite for functionality of the peptides. Loosely bound RGD or RGD present in solution actually inhibits adhesion.^6^, ^11^ The proper attachment of both endothelial cells and smooth muscle cells in this study on the RGD functionalized material, alone and in combination with the antifouling BOB additive, shows that our supramolecular system allows for proper anchoring of peptide ligands of similar design. Surprisingly, the non‐integrin binding SDF1α peptide did show refunctionalization capability. It is known that cell‐binding is not necessarily integrin mediated,^76^ but impaired focal adhesion formation may result in altered cellular morphology, just as was observed here for the SDF1α functionalized surfaces. Besides proper incorporation, grafting density and distribution of biomimetic adhesive ligands can influence their efficacy. For RGD grafting densities as low as 1 fmol/cm^2^ have been reported to induce cell adhesion and spreading^77^ and for REDV a density of 10 pmol/cm^2^ was reported.^10^ The additives and base material are self‐assembled into structures described here, and therefore the peptide concentration at the surface is unknown and would be particularly hard to quantify. However, in a system with mesoscopically similar assembly that relies on a quadruple hydrogen bonding motif, an additive with a OEG_6_ spacer was shown to accumulate at the surface of the supramolecular material.^78^ Theoretically, if all the incorporated peptide for 4 mol% BU‐peptide is present at the surface, a density of approximately 10 nmol/cm^2^ is obtained. Note that, in the unlikely scenario where only a fraction of the incorporated additive is available at the surface, the density would still be relatively high.^38^ Comparing the affinity to different peptide‐functionalized surfaces is non‐trivial, since the chemistry used to tether the peptide to a material, and the amino‐acids that flank the principal sequence can influence this affinity. The threshhold for cell‐spreading has been shown to be 6x higher for REDV when compared to RGD.^79^ This suggested difference in ligand density required for effective functionalization with REDV, compared to RGD, may indicate a difference in affinity of integrins binding to these receptors. Moreover, half of the integrins is able to bind RGD motifs,^6^ whereas REDV binding relies on the α4β1 integrin. Nonetheless, inconsistent results have been reported for the efficacy of REDV functionalization. Some studies report improved adhesion of endothelial cells but do not compare to RGD or other peptides for functionalization,^14^, ^15^, ^16^, ^19^, ^20^, ^21^, ^22^, ^25^, ^26^, ^27^, ^29^, ^32^, ^33^, ^34^, ^35^, ^36^, ^37^, ^39^, ^80^, ^81^ or adhesion of other mammalian cell types.,^15^, ^18^, ^19^, ^20^, ^24^, ^26^, ^27^, ^29^, ^32^, ^36^, ^37^, ^73^, ^82^ or show unexpected failed refunctionalization for other cell‐types with RGD.^10^ Other studies also unexpectedly show reduced endothelial cell adhesion on REDV functionalized surfaces compared to RGD.^30^, ^38^, ^73^, ^82^, ^83^, ^84^

Furthermore, the supramolecular interactions, which are the driving force behind the functionalization strategy applied here, add complexity. Small changes in the molecular structure of the peptide influence the intermolecular interactions and the assembly of the peptide‐additives at the surface. Since availability of the peptide for binding with integrins is essential, and the assembly state can influence the functionality of peptide‐based supramolecular additives,^85^ the availability of the peptides in a self‐assembled state should be investigated further and may provide tools for improved rational design of functional peptide additives. Yet, taking into account the concerns raised above, the comparison between different biomaterial platforms, with different functionalization chemistries, ligand densities, and experimental methods remains a challenge.

## CONCLUSIONS

These results serve as a reminder that in the design and fabrication of biomimetic materials we should heed to capture the complexity that is found in nature. Ligand chemical nano‐environment, spacing, ordening, density, and affinity all influence the cellular responses in both natural and synthetic systems. Here we have demonstrated in a combinatorial non‐covalent approach that a supramolecular mixture of a polymeric base material, non‐cell adhesive BOB, and the generic BU‐peptide conjugate BU‐RGD results in re‐activated and cell adhesive surface. However, the supramolecular incorporation of BU‐REDV showed little activity, whereas the BU‐SDF1α exhibited aspecific functionality, both alone and in combined formulation with the BOB. These results were hard to correlate to physical and chemical properties of these surfaces, and the chemical nano‐environment of the peptides, which highlights that care should be taken with the predictive value of such read‐outs for future screening purposes.

## Supporting information


**Appendix S1**: Supporting Information

## References

[1] J. A. Hubbell , Curr. Opin. Biotechnol. 1999, 10, 123. 10209141 10.1016/s0958-1669(99)80021-4

[2] H. Shin , S. Jo , A. G. Mikos , Biomaterials 2003, 24, 4353. 12922148 10.1016/s0142-9612(03)00339-9

[3] Wilson, C. J. , Clegg, R. E. , Leavesley, D. I. , Pearcy, M. J. , Tissue Eng. 2005, 11, 1. 15738657 10.1089/ten.2005.11.1

[4] Goddard, J. M. , Hotchkiss, J. H.Ã. 2007, Prog. Polym. Sci. 32, 698.

[5] M. D. Pierschbacher , E. Ruoslahti , Nature 1984, 309, 30. 6325925 10.1038/309030a0

[6] U. Hersel , C. Dahmen , H. Kessler , Biomaterials 2003, 24, 4385. 12922151 10.1016/s0142-9612(03)00343-0

[7] L. Perlin , S. MacNeil , S. Rimmer , Soft Matter 2008, 4, 2331.

[8] G. Delaittre , A. M. Greiner , T. Pauloehrl , C. Barner‐kowollik , D. P. J. Verne , M. Bastmeyer , C. Barner‐kowollik , Soft Matter 2012, 8, 7323.

[9] M. B. Rahmany , M. van Dyke , Acta Biomater. 2013, 9, 5431. 23178862 10.1016/j.actbio.2012.11.019

[10] J. A. Hubbell , S. P. Massia , N. P. Desai , P. D. Drumheller , Nat. Biotechnol. 1991, 9, 568. 10.1038/nbt0691-568 1369319

[11] S. P. Massia , J. A. Hubbell , J. Biol. Chem. 1992, 267, 14019. 1629200

[12] A. P. Mould , A. Komoriya , K. M. Yamada , M. J. Humphries , J. Biol. Chem. 1991, 266, 3579. 1750929

[13] C. H. Park , Y. J. Hong , K. Park , D. K. Han , Macromol. Res. 2010, 18, 526.

[14] Y. Ji , Y. Wei , X. Liu , J. Wang , K. Ren , J. Ji , J. Biomed. Mater. Res.—Part A 2012, 100, 1387. 10.1002/jbm.a.34077 22374807

[15] S. Kakinoki , T. Yamaoka , Bioconjug. Chem. 2015, 26, 639. 25742028 10.1021/acs.bioconjchem.5b00032

[16] W. Zhan , X. Shi , Q. Yu , Z. Lyu , L. Cao , H. Du , Q. Liu , X. Wang , G. Chen , D. Li , J. L. Brash , H. Chen , Adv. Funct. Mater. 2015, 25, 5206.

[17] Y. Wei , J. Zhang , Xun , Y. Ji , J. Ji , Colloids Surf. B Biointerf. 2015, 136, 1166. 10.1016/j.colsurfb.2015.11.012 26613858

[18] F. Zhou , X. Jia , Y. Yang , Q. Yang , C. Gao , Y. Zhao , Y. Fan , X. Yuan , Mater. Sci. Eng. C 2016, 68, 623. 10.1016/j.msec.2016.06.036 27524062

[19] M. Gabriel , K. Niederer , M. Becker , C. M. Raynaud , C. F. Vahl , H. Frey , Bioconjug. Chem. 2016, 27, 1216. 27041509 10.1021/acs.bioconjchem.6b00047

[20] B. a. Butruk‐Raszeja , M. S. Dresler , A. Kumiska , T. Ciach , Colloids Surf. B Biointerf. 2016, 144, 335. 10.1016/j.colsurfb.2016.04.017 27110909

[21] S. Yu , Y. Gao , X. Mei , T. Ren , S. Liang , Z. Mao , C. Gao , ACS Appl. Mater. Interfaces 2016, 8, 29280. 27723284 10.1021/acsami.6b09375

[22] Y. Wei , J. Zhang , X. Feng , D. Liu , J. Biomater. Sci. Polym. Ed. 2017, 28, 2101. 28891389 10.1080/09205063.2017.1376829

[23] M. I. Castellanos , C. Mas‐Moruno , A. Grau , X. Serra‐Picamal , X. Trepat , F. Albericio , M. Joner , F. J. Gil , M. P. Ginebra , J. M. Manero , M. Pegueroles , Appl. Surf. Sci. 2017, 393, 82.

[24] Y. Kambe , A. Murakoshi , H. Urakawa , Y. Kimura , T. Yamaoka , J. Mater. Chem. B 2017, 5, 7557. 10.1039/c7tb02109g 32264231

[25] Q. K. Lin , Y. Hou , K. F. Ren , J. Ji , Thin Solid Films 2012, 520, 4971.

[26] Z. Li , P. Zhou , F. Zhou , Y. Zhao , L. Ren , X. Yuan , Colloids Surf. B Biointerf. 2018, 162, 335. 10.1016/j.colsurfb.2017.12.004 29223648

[27] S. Kakinoki , K. Takasaki , A. Mahara , T. Ehashi , Y. Hirano , T. Yamaoka , J. Biomed. Mater. Res.—Part A 2018, 106, 491. 10.1002/jbm.a.36258 28975703

[28] X. Ding , W. Chin , C. N. Lee , J. L. Hedrick , Y. Y. Yang , Adv. Healthc. Mater. 2018, 7, 1. 10.1002/adhm.201700944 29205938

[29] F. Zhou , M. Wen , P. Zhou , Y. Zhao , X. Jia , Y. Fan , X. Yuan , Mater. Sci. Eng. C 2018, 85, 37. 10.1016/j.msec.2017.12.005 29407155

[30] B. Colak , S. Di Cio , J. E. Gautrot , S. di Cio , J. E. Gautrot , Biomacromolecules 2018, 19, 1445. 29294284 10.1021/acs.biomac.7b01436

[31] T. Flora , I. G. de Torre , L. Quintanilla , M. Alonso , J. C. Rodríguez‐Cabello , Eur. Polym. J. 2018, 106, 19.

[32] J. Devalliere , Y. Chen , K. Dooley , M. L. Yarmush , B. E. Uygun , Acta Biomater. 2018, 78, 151. 30071351 10.1016/j.actbio.2018.07.046 PMC6261340

[33] Y. Wei , Y. Ji , L.‐L. Xiao , Q. Lin , J. Xu , K. Ren , J. Ji , Biomaterials 2013, 34, 2588. 23352039 10.1016/j.biomaterials.2012.12.036

[34] Y. Liu , T. T. Yang Tan , S. Yuan , C. Choong , J. Mater. Chem. B 2013, 1, 157. 10.1039/c2tb00014h 32260688

[35] J. Yang , M. Khan , L. Zhang , X. Ren , J. Guo , Y. Feng , S. Wei , W. Zhang , J. Mater. Chem. B 2015, 3, 7682. 10.1039/c5tb01155h 32264578

[36] S. Yuan , G. Xiong , F. He , W. Jiang , B. Liang , C. Choong , J. Mater. Chem. B 2015, 3, 8088. 10.1039/c5tb01598g 32262866

[37] M. I. Castellanos , A. S. Zenses , A. Grau , J. C. Rodríguez‐Cabello , F. J. Gil , J. M. Manero , M. Pegueroles , Colloids Surf. B Biointerf. 2015, 127, 22. 10.1016/j.colsurfb.2014.12.056 25637794

[38] S. Noel , A. Hachem , Y. Merhi , G. de Crescenzo , Biomacromolecules 2015, 16, 1682. 25877934 10.1021/acs.biomac.5b00219

[39] Y. Wang , S. Chen , Y. Pan , J. Gao , D. Tang , D. Kong , S. Wang , J. Mater. Chem. B 2015, 3, 9212. 10.1039/c5tb02080h 32263136

[40] D. E. P. Muylaert , G. C. van Almen , H. Talacua , J. O. Fledderus , J. Kluin , S. I. S. Hendrikse , J. L. J. van Dongen , E. Sijbesma , A. W. Bosman , T. Mes , S. H. Thakkar , A. I. P. M. Smits , C. V. C. Bouten , P. Y. W. Dankers , M. C. Verhaar , Biomaterials 2016, 76, 187. 26524538 10.1016/j.biomaterials.2015.10.052

[41] S. P. Massia , J. Cell Biol. 2004, 114, 1089. 10.1083/jcb.114.5.1089 PMC2289117 1714913

[42] X. Han , Y. Liu , F. G. Wu , J. Jansensky , T. Kim , Z. Wang , C. L. Brooks , J. Wu , C. Xi , C. M. Mello , Z. Chen , J. Phys. Chem. B 2014, 118, 2904. 24555411 10.1021/jp4122003

[43] M. J. Wilson , S. J. Liliensiek , C. J. Murphy , W. L. Murphy , P. F. Nealey , Soft Matter 2012, 8, 390. 23264803 10.1039/C1SM06589K PMC3526380

[44] S. Sur , F. Tantakitti , J. B. Matson , S. I. Stupp , Biomater. Sci. 2015, 3, 520. 26222295 10.1039/c4bm00326h

[45] J. H. Beer , K. T. Springer , B. S. Coller , Blood 1992, 79, 117. 1728303

[46] S. J. Attwood , E. Cortes , A. W. M. Haining , B. Robinson , D. Li , J. Gautrot , A. del Río Hernández , Sci. Rep. 2016, 6, 34334. 27686622 10.1038/srep34334 PMC5043376

[47] B. T. Houseman , M. Mrksich , Biomaterials 2001, 22, 943. 11311013 10.1016/s0142-9612(00)00259-3

[48] D. Grafahrend , K.‐H. Heffels , M. V. Beer , P. Gasteier , M. Möller , G. Boehm , P. D. Dalton , J. Groll , Nat. Mater. 2011, 10, 67. 21151163 10.1038/nmat2904

[49] A. E. Rodda , F. Ercole , V. Glattauer , J. Gardiner , D. R. Nisbet , K. E. Healy , J. S. Forsythe , L. Meagher , Biomacromolecules 2015, 16, 2109. 26020464 10.1021/acs.biomac.5b00483

[50] S. C. Lange , E. van Andel , M. M. J. Smulders , H. Zuilhof , Langmuir 2016, 32, 10199. 27687696 10.1021/acs.langmuir.6b02622

[51] B. D. Ippel , P. Y. W. Dankers , Adv. Healthc. Mater. 2018, 7, 1700505. 10.1002/adhm.201700505 28841771

[52] O. J. G. M. Goor , S. I. S. Hendrikse , P. Y. W. Dankers , E. W. Meijer , Chem. Soc. Rev. 2017, 46, 6621. 28991958 10.1039/c7cs00564d

[53] T. Aida , E. W. Meijer , S. I. Stupp , Science (80‐. ) 2012, 335, 813. 10.1126/science.1205962 PMC3291483 22344437

[54] S. Spaans , P. P. K. H. Fransen , B. D. Ippel , D. F. A. de Bont , H. M. Keizer , N. A. M. A. M. Bax , C. V. C. Bouten , P. Y. W. Dankers , Biomater. Sci. 2017, 5, 1541. 28636048 10.1039/c7bm00407a

[55] H. Storrie , M. O. Guler , S. N. Abu‐Amara , T. Volberg , M. Rao , B. Geiger , S. I. Stupp , Biomaterials 2007, 28, 4608. 17662383 10.1016/j.biomaterials.2007.06.026

[56] P. Y. W. Dankers , M. C. Harmsen , L. A. Brouwer , M. J. A. van Luyn , E. W. Meijer , Nat. Mater. 2005, 4, 568. 15965478 10.1038/nmat1418

[57] R. C. van Gaal , A. B. C. Buskermolen , B. D. Ippel , P.‐P. K. H. Fransen , S. Zaccaria , C. V. C. Bouten , P. Y. W. Dankers , Biomaterials 2019, 224, 119466. 31542516 10.1016/j.biomaterials.2019.119466

[58] J. Kluin , H. Talacua , A. I. P. M. P. M. Smits , M. Y. Emmert , M. C. P. Brugmans , E. S. Fioretta , P. E. Dijkman , S. H. M. Söntjens , R. Duijvelshoff , S. Dekker , M. W. J. T. Janssen‐van den Broek , V. Lintas , A. Vink , S. P. Hoerstrup , H. M. Janssen , P. Y. W. W. Dankers , F. P. T. T. Baaijens , C. V. C. C. Bouten , Biomaterials 2017, 125, 101. 28253994 10.1016/j.biomaterials.2017.02.007

[59] X. Gu , Y. Matsumura , Y. Tang , S. Roy , R. Hoff , B. Wang , W. R. Wagner , Biomaterials 2017, 133, 132. 28433936 10.1016/j.biomaterials.2017.04.015

[60] Y. Hong , S. H. Ye , A. Nieponice , L. Soletti , D. A. Vorp , W. R. Wagner , Biomaterials 2009, 30, 2457. 19181378 10.1016/j.biomaterials.2009.01.013 PMC2698791

[61] R. Duijvelshoff , N. van Engeland , K. Gabriels , S. Söntjens , A. Smits , P. Dankers , C. Bouten , Bioengineering 2018, 5, 61. 10.3390/bioengineering5030061 PMC6164451 30082586

[62] A. M. Seifalian , H. J. Salacinski , A. Tiwari , A. Edwards , S. Bowald , G. Hamilton , Biomaterials 2003, 24, 2549. 12695082 10.1016/s0142-9612(02)00608-7

[63] R. A. Koevoets , R. M. Versteegen , H. Kooijman , A. L. Spek , R. P. Sijbesma , E. W. Meijer , J. Am. Chem. Soc. 2005, 127, 2999. 15740137 10.1021/ja0451160

[64] E. Wisse , A. J. H. Spiering , E. N. M. van Leeuwen , R. A. E. Renken , P. Y. W. Dankers , L. A. Brouwer , M. J. A. van Luyn , M. C. Harmsen , N. A. J. M. Sommerdijk , E. W. Meijer , Biomacromolecules 2006, 7, 3385. 17154467 10.1021/bm060688t

[65] N. E. Botterhuis , S. Karthikeyan , D. Veldman , S. C. J. Meskers , R. P. Sijbesma , Chem. Commun. 2008, 33, 3915. 10.1039/b804457k 18726033

[66] N. E. Botterhuis , S. Karthikeyan , A. J. H. Spiering , R. P. Sijbesma , Macromolecules 2010, 43, 745.

[67] B. D. Ippel , H. M. Keizer , P. Y. W. Dankers , Adv. Funct. Mater. 2019, 29, 1805375.

[68] S. Zaccaria , R. C. van Gaal , M. Riool , S. A. J. Zaat , P. Y. W. Dankers , J. Polym. Sci. Part A Polym. Chem. 2018, 56, 1926. 10.1002/pola.29078 PMC6175361 30344368

[69] B. B. Mollet , M. Comellas‐Aragonès , A. J. H. Spiering , S. H. M. Söntjens , E. W. Meijer , P. Y. W. Dankers , J. Mater. Chem. B 2014, 2, 2483. 10.1039/c3tb21516d 32261418

[70] A. M. Castagna , A. Pangon , G. P. Dillon , J. Runt , Macromolecules 2013, 46, 6520.

[71] V. Bonito , A. I. P. M. Smits , O. J. G. M. Goor , B. D. Ippel , A. Driessen‐Mol , T. J. A. G. Münker , A. W. Bosman , T. Mes , P. Y. W. Dankers , C. V. C. Bouten , Acta Biomater. 2018, 71, 1. 29518556 10.1016/j.actbio.2018.02.032

[72] S. Chen , L. Li , C. Zhao , J. Zheng , Polymer (Guildf). 2010, 51, 5283.

[73] Y. Lei , M. Remy , C. Labrugere , M. C. Durrieu , J. Mater. Sci. Mater. Med. 2012, 23, 2761. 22878726 10.1007/s10856-012-4736-x

[74] M. V. Volin , L. Joseph , M. S. Shockley , P. F. Davies , Biochem. Biophys. Res. Commun. 1998, 242, 46. 9439607 10.1006/bbrc.1997.7890

[75] Q. Yu , Y. Zhang , H. Wang , J. Brash , H. Chen , Acta Biomater. 2011, 7, 1550. 21195214 10.1016/j.actbio.2010.12.021

[76] S. M. Albelda , C. A. Buck , FASEB J. 1990, 4, 2868. 2199285

[77] Massia, S. P. , Hubbell, J. A. , J. Cell Biol. 1991, 114, 1089. 1714913 10.1083/jcb.114.5.1089 PMC2289117

[78] O. J. G. M. Goor , H. M. Keizer , A. L. Bruinen , M. G. J. Schmitz , R. M. Versteegen , H. M. Janssen , R. M. A. Heeren , P. Y. W. Dankers , Adv. Mater. 2017, 29, 1604652. 10.1002/adma.201604652 27896852

[79] Mould, A. P. , Komoriyae, A. , Yamadall, K. M. , Humphriess, M. J. , J. Biol. Chem. 1991, 266, 3579. 1750929

[80] Y. Wei , J. Zhang , H. Li , L. Zhang , H. Bi , J. Biomater. Sci. Polym. Ed. 2015, 26, 1357. 26381476 10.1080/09205063.2015.1095024

[81] Y. Wei , Y. Ji , L. Xiao , Q. Lin , J. Ji , Colloids Surfaces B Biointerfaces 2011, 84, 369. 21333506 10.1016/j.colsurfb.2011.01.028

[82] I. G. De Torre , F. Wolf , M. Santos , L. Rongen , M. Alonso , S.,. C. Jockenhoevel , J. Rodríguez‐Cabello , P. Mela , Acta Biomater. 2015, 12, 146. 25448343 10.1016/j.actbio.2014.10.029

[83] S. C. Heilshorn , K. A. DiZio , E. R. Welsh , D. A. Tirrell , Biomaterials 2003, 24, 4245. 12853256 10.1016/s0142-9612(03)00294-1

[84] J. P. Jung , J. V. Moyano , J. H. Collier , Integr. Biol. 2011, 3, 185. 10.1039/c0ib00112k PMC3401080 21249249

[85] M. Putti , O. M. J. A. Stassen , M. J. G. Schotman , C. M. Sahlgren , P. Y. W. Dankers , ACS Omega 2019, 4, 8178. 31172036 10.1021/acsomega.9b00869 PMC6545632

