# Supplementary material for: Combinatorial functionalization with bisurea‐peptides and antifouling bisurea additives of a supramolecular elastomeric biomaterial
Source: J Polym Sci B Polym Phys. 2019 Dec 3;57(24):1725–35. doi: 10.1002/polb.24907 (PMC6988465; doi:10.1002/polb.24907)
Supplement: Supplementary file 1 — Appendix S1: Supporting Information [file POLB-57-1725-s001.pdf]

SUPPORTING INFORMATION - Combinatorial functionalization with bisurea-peptides and antifouling bisurea additives of a supramolecular elastomeric biomaterial

Bastiaan D. Ippel,[1,2] Boris Arts,[1,3] Henk M. Keizer,[4] Patricia Y.W. Dankers[1,2,3]

1. Institute for Complex Molecular Systems, Eindhoven University of Technology, PO Box 513 5600 MB, Eindhoven, The Netherlands
2. Laboratory for Cell and Tissue Engineering, Eindhoven University of Technology, PO Box 513 5600 MB, Eindhoven, The Netherlands
3. Laboratory for Chemical Biology, Eindhoven University of Technology, PO Box 513, 5600 MB, Eindhoven, The Netherlands
4. SyMO-Chem B.V., Den Dolech 2, 5612 AZ, Eindhoven, The Netherlands

Synthesis of bisurea carboxylic acid precursor

Phenyl (4-(3-hexylureido)butyl)carbamate (1)

Butane-1,4-diisocyanate (1.82 mL, 14.3 mmol) and phenol (1.35 g, 14.3 mmol) were stirred at room temperature in 50 mL dichloromethane until dissolved. DIPEA (5 mL, 28.8 mmol) was added to the reaction mixture, resulting in the formation of a white dispersion. After stirring for 1 hour, hexylamine (1.95 mL, 14.7 mmol) was added to the mixture, resulting in the formation of a precipitate that made stirring difficult. The reaction mixture was subsequently diluted with 25 mL dichloromethane and stirred for another 30 minutes, after which FT-IR showed complete conversion of the isocyanate. The white precipitate was collected by filtration over a Büchner funnel, flushed with 50 mL dichloromethane and dried to afford 3.9 g crude product. This product was stirred with 200 mL 1:9 methanol/chloroform and the resulting suspension was filtered over diatomaceous earth. The plug was flushed with an additional 100 mL of the 1:9 methanol/chloroform mixture, after which the filtrates were combined and evaporated to dryness. The resulting crude product was further purified by column chromatography (SiO_2, 5:95 MeOH/$CHCl_3$) to afford 1.79 g (75%) of the desired building block **1**.

^{1}H-NMR (400 MHz, DMSO-d6): δ = 7.74 (t, 1H), 7.37 (m, 2H), 7.19 (m, 1H), 7.08 (m, 2H), 5.74 (m, 2H), 3.20-2.85 (br.m, 6H), 1.60-1.16 (br.m, 12H), 0.86 (t, 3H) ppm. ^{13}C-NMR (101 MHz, DMSO-d_6): δ = 158.1, 154.3, 151.1, 129.2, 124.8, 121.7, 40.3, 39.2, 38.9, 31.0, 30.0, 27.5, 26.8, 26.1, 22.1, 13.9 ppm. FT-IR (ATR): ν = 3388, 3255, 3056, 2953, 2929, 2863, 1710, 1648, 1557, 1534, 1498, 1475, 1456, 1376, 1275, 1235, 1208, 1120, 1072, 1038, 1025, 990, 781, 747, 716, 682, 603, 505 cm^{-1}. MALDI-TOF-MS (DCTB matrix): calculated for $C_{18}H_{29}N_3O_3$: 335.22 Da, found: 336.28 $[M+H]^+$, 358.26 $[M+Na]^+$ Da. LC-MS(ESI) R_t= 6.37 min m/z calcd ($C_{18}H_{29}N_3O_3$) 335.2; found 242.3 $[M\text{-phenoxy}+H]^+$, 336.2 $[M+H]^+$, 358.3 $[M+Na]^+$, 671.0 $[2M+H]^+$, 693.0 $[2M+Na]^+$.

t-Butyl-41,56,63-trioxo-4,7,10,13,16,19,22,25,28,31,34,37,40-tridecaoxa-42,55,57,62,64-pentaazaheptacontanoate; C6-U4U-C12-dPEG12-C2-COOtBu (2)

The amine NH_2-C12-dPEG12-C2-COO*t*Bu, which was synthesized as described previously[1], (650 mg, 0.72 mmol) and building block **1** (297 mg, 0.89 mmol) were dissolved in 10 mL DMF, followed by the addition of DBU base (0.44 mL, 1.29 mmol). The reaction mixture was stirred for 3 hours at 50°C under an atmosphere of argon. Chloroform (50 mL) was added, the organic phase was washed with 0.5 M citric acid (2x) and brine (2x), and was then dried over Na_2SO_4. Concentration of the solution was followed by stirring of the solid residue in ether, and decanting of the superfluent. The solid was purified by elution over silica using MeOH/$CHCl_3$ 4/96 affording 0.52 g (63 %) of product **2**.

^{1}H NMR (400 MHz, $CDCl_3$): δ 5.75 – 5.23 (m, 1H, NH), 4.19 (t, *J* = 4.7 Hz, 2H), 4.01 (t, *J* = 5.7 Hz, 14H, H_2O), 3.65 (s, 48H), 3.37 (d, *J* = 2.9 Hz, 1H), 3.11 (q, *J* = 7.3, 6.6 Hz, 10H), 2.51 (t, *J* = 6.5 Hz, 2H), 1.55 – 1.38 (m, 19H), 1.29 (dd, *J* = 13.5, 8.3 Hz, 22H), 0.95 – 0.80 (m, 3H). ^{13}C NMR (101 MHz, $CDCl_3$): δ = 171.1, 159.0, 156.6, 80.7, 70.7, 70.7, 70.6, 70.5, 69.8, 67.0, 63.9, 41.2, 40.6, 40.6, 40.0, 36.4, 31.7, 30.5, 30.0, 29.6, 29.5, 29.4, 29.3, 28.2, 27.8, 27.7, 27.0, 26.8, 26.8, 22.7, 14.2 ppm. FT-IR (ATR): ν = 3325, 2923, 2856, 1726, 1615, 1580, 1536, 1477, 1457, 1367, 1350, 1251, 1106, 950, 848, 776, 751, 627, 586 cm^{-1}. HPLC-MS(ESI) R_t= 8.29 min m/z calcd ($C_{56}H_{111}N_5O_{18}$) 1142.5; found 543.8 $[M\text{-}tBu+2H]^{2+}$, 582.9 $[M+H+Na]^{2+}$, 594.0 $[M+2Na]^{2+}$, 1142.5 $[M+H]^+$, 1164.8 $[M+Na]^+$.

41,56,63-Trioxo-4,7,10,13,16,19,22,25,28,31,34,37,40-tridecaoxa-42,55,57,62,64-pentaazaheptacontanoic acid; C6-U4U-C12-dPEG12-C2-COOH (3)

To a solution of C6-U4U-C12-dPEG12-C2-COOtBu **2** (0.52 g, 0.46 mmol) in DCM (5 mL), TFA (5 mL, 65 mmol) was added and the clear solution was stirred for 1 hour under an argon atmosphere. The solvent was removed under reduced pressure. The residue was co-evaporated twice with toluene to remove traces of TFA. Ether (15 mL) was added, the suspension was stirred, and the superfluent was decanted off (2x). The residue was dissolved in a small volume of $CHCl_3$/MeOH 90/10 and the resulting solution was pipetted into ether. The precipitate was collected by centrifugation, and was dried, affording product **3** (0.40 g, 91%) as a waxy solid.

^{1}H NMR (400 MHz, $CDCl_3$): δ 5.38 (dt, *J* = 74.3, 5.9 Hz, 1H), 4.27 – 4.13 (m, 2H), 3.76 (t, *J* = 6.4 Hz, 2H), 3.64 (d, *J* = 6.1 Hz, 44H), 3.53 – 3.29 (m, 19H, H_2O), 3.11 (q, *J* = 7.5, 7.0 Hz, 10H), 2.59 (t, *J* = 6.4 Hz, 2H), 1.46 (p, *J* = 7.1, 6.6 Hz, 10H), 1.28 (dd, *J* = 11.4, 5.9 Hz, 22H), 0.96 – 0.82 (m, 3H). ^{13}C NMR (101 MHz, $CDCl_3$): δ 173.76, 159.44, 156.69, 70.37, 69.52, 66.59, 63.66, 49.71, 49.49, 49.28, 49.06, 48.85, 48.64, 48.42, 40.80, 40.18, 40.06, 39.51, 39.38, 34.78, 31.45, 30.08, 30.03, 29.73, 29.45, 29.42, 29.26, 29.16, 27.14, 26.79, 26.64, 26.46, 22.47, 13.86. FT-IR (ATR): ν = 3323, 2922, 2852, 1727, 1680, 1616, 1577, 1539, 1477, 1466, 1345, 1268, 1236, 1194, 1105, 948, 842, 777, 750, 724, 624, 585, 410 cm^{-1}. HPLC-MS(ESI) R_t= 6.52 min m/z calcd ($C_{52}H_{103}N_5O_{18}$) 1086.4; found 543.9 $[M+2H]^{2+}$, 555.0 $[M+H+Na]^{2+}$, 566.1 $[M+2Na]^{2+}$, 1086.8 $[M]^+$, 1109.0 $[M+Na]^+$, 1130.8 $[M-H+2Na]^+$.

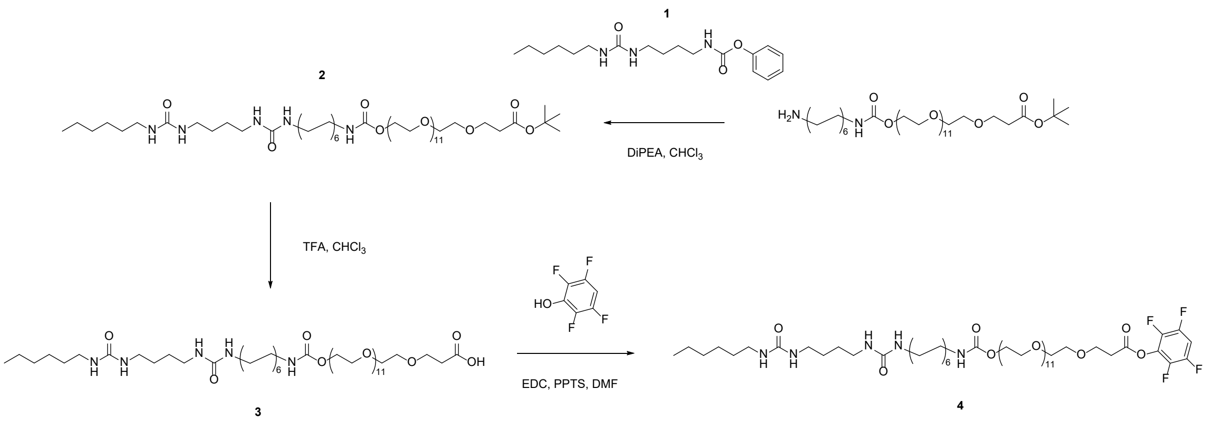

Scheme S1 Synthesis route for bisurea-COOH (**3**) and bisurea-TFP-ester (**4**)

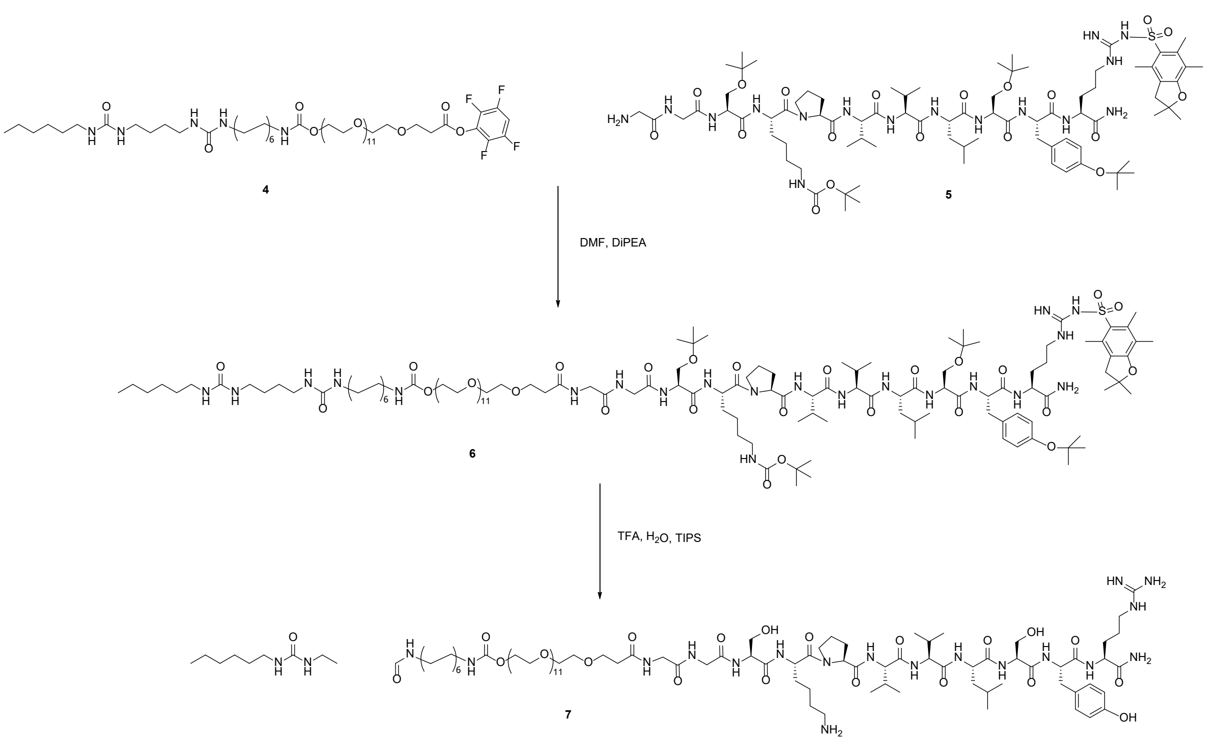

Scheme S2 Coupling of protected SDF1α-peptide to bisurea-TFP-ester and subsequent deprotection.

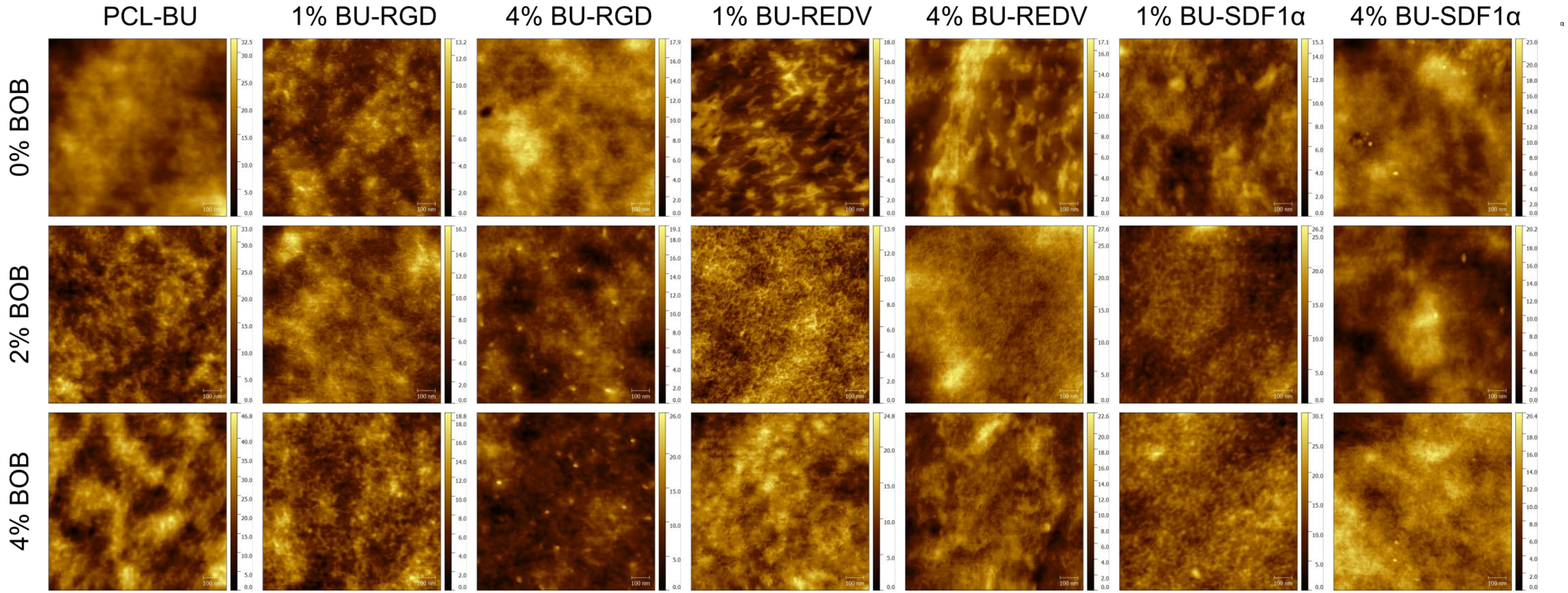

FIGURE S1 Atomic Force Microscopy height images recorded in tapping mode in air of solution-cast films of PCL-BU with mixtures of BU-OEG-BU and the BU-peptide conjugates. Scale bars represent 100 nm.

TABLE S1 RMS roughness in nm of solution-cast films of PCL-BU with mixtures of BU-OEG-BU and the BU-peptide additives.

	**PCL-BU**	**1% BU-RGD**	**4% BU-RGD**	**1% BU-REDV**	**4% BU-REDV**	**1% BU-SDF1α**	**4% BU-SDF1α**
**0% BOB**	3.95	1.56	2.08	2.51	2.68	1.83	2.76
**2% BOB**	4.26	2.08	2.16	1.74	3.41	3.02	2.97
**4% BOB**	7.37	2.49	2.61	3.25	2.86	3.47	2.76

TABLE S2 Elemental Composition of surface of solution-cast films of PCL-BU functionalized with mixtures of BOB and BU-peptide conjugates

**Surface**	Surface Composition [at %]			Ratio
	**C**	**O**	**N**	**C/N**
PCL-BU	77.4	21.43	1.17	66.2
+ 2% BU-OEG-BU	75.2	22.6	2.16	34.8
+ 4% BU-OEG-BU	73.2	20.2	6.6	11.1
+ 1% BU-RGD	73.09	22.12	4.79	15.3
+ 4% BU-RGD	70.44	22.03	7.54	9.3
+ 1% BU-REDV	74.14	22.03	3.83	19.4
+ 4% BU-REDV	70.65	21.76	7.59	9.3
+ 1% BU-SDF1α	73.2	21.71	5.09	14.4
+ 4% BU-SDF1α	71.56	21.13	7.31	9.8
+ 2% BU-OEG-BU + 1% BU-RGD	72.9	21.98	5.13	14.2
+ 4% BU-OEG-BU +1% BU-RGD	75.28	21.84	2.88	26.1
+ 2% BU-OEG-BU + 4% BU-RGD	71.64	21.73	6.63	10.8
+ 4% BU-OEG-BU + 4% BU-RGD	72.58	21.66	5.76	12.6
+ 2% BU-OEG-BU + 1% BU-REDV	73.67	21.53	4.8	15.3
+ 4% BU-OEG-BU + 1% BU-REDV	72.54	21.23	6.23	11.6
+ 2% BU-OEG-BU + 4% BU-REDV	70.68	21.5	7.82	9.0
+ 4% BU-OEG-BU + 4% BU-REDV	70.71	21.33	7.96	8.9
+ 2% BU-OEG-BU + 1% BU-SDF1α	73.47	21.48	5.06	14.5
+ 4% BU-OEG-BU + 1% BU-SDF1α	72.95	20.87	6.18	11.8
+ 2% BU-OEG-BU + 4% BU-SDF1α	71.04	21.03	7.92	9.0
+ 4% BU-OEG-BU + 4% BU-SDF1α	71.24	20.74	8.02	8.9

TABLE S3 Quantification of components in XPS C1s narrow scan of solution-cast films of PCL-BU functionalized with mixtures of BOB and BU-peptide conjugates

**Surface**	Carbon species				
	**C-C**	**C-O**	**C-N**	**O=C-N**	**O=C-O**
PCL-BU	65.17	18.05	1.05	1.12	14.61
+ 2% BU-OEG-BU	61.17	22.53	2.11	1.33	12.86
+ 4% BU-OEG-BU	45.01	36.69	7.64	3.31	7.35
+ 1% BU-RGD	51.84	24.18	7.85	4.13	12
+ 4% BU-RGD	42.5	29.04	12.11	7.07	9.27
+ 1% BU-REDV	57.14	20.62	5.78	2.7	13.75
+ 4% BU-REDV	44.32	27.49	11.29	7.56	9.34
+ 1% BU-SDF1α	54.2	22.53	7.73	4.34	11.2
+ 4% BU-SDF1α	48.29	27.22	9.33	7.22	7.95
+ 2% BU-OEG-BU + 1% BU-RGD	47.82	29.02	8.98	4.1	10.08
+ 4% BU-OEG-BU +1% BU-RGD	52.49	26.6	7.21	2.27	11.43
+ 2% BU-OEG-BU + 4% BU-RGD	42.52	31.61	11.2	6.03	8.64
+ 4% BU-OEG-BU + 4% BU-RGD	43.18	33.85	9.44	5.49	8.05
+ 2% BU-OEG-BU + 1% BU-REDV	51.12	25.96	8.35	3.5	11.06
+ 4% BU-OEG-BU + 1% BU-REDV	43.65	35.81	8.58	4.45	7.51
+ 2% BU-OEG-BU + 4% BU-REDV	43.26	30.09	11.02	7.15	8.47
+ 4% BU-OEG-BU + 4% BU-REDV	41.94	32.8	10.7	6.77	7.8
+ 2% BU-OEG-BU + 1% BU-SDF1α	51.46	25.64	8.29	4.24	10.37
+ 4% BU-OEG-BU + 1% BU-SDF1α	45.99	32.66	8.76	4.44	8.15
+ 2% BU-OEG-BU + 4% BU-SDF1α	45.93	30.47	9.37	7.32	6.91
+ 4% BU-OEG-BU + 4% BU-SDF1α	45.17	32.42	8.9	6.95	6.55

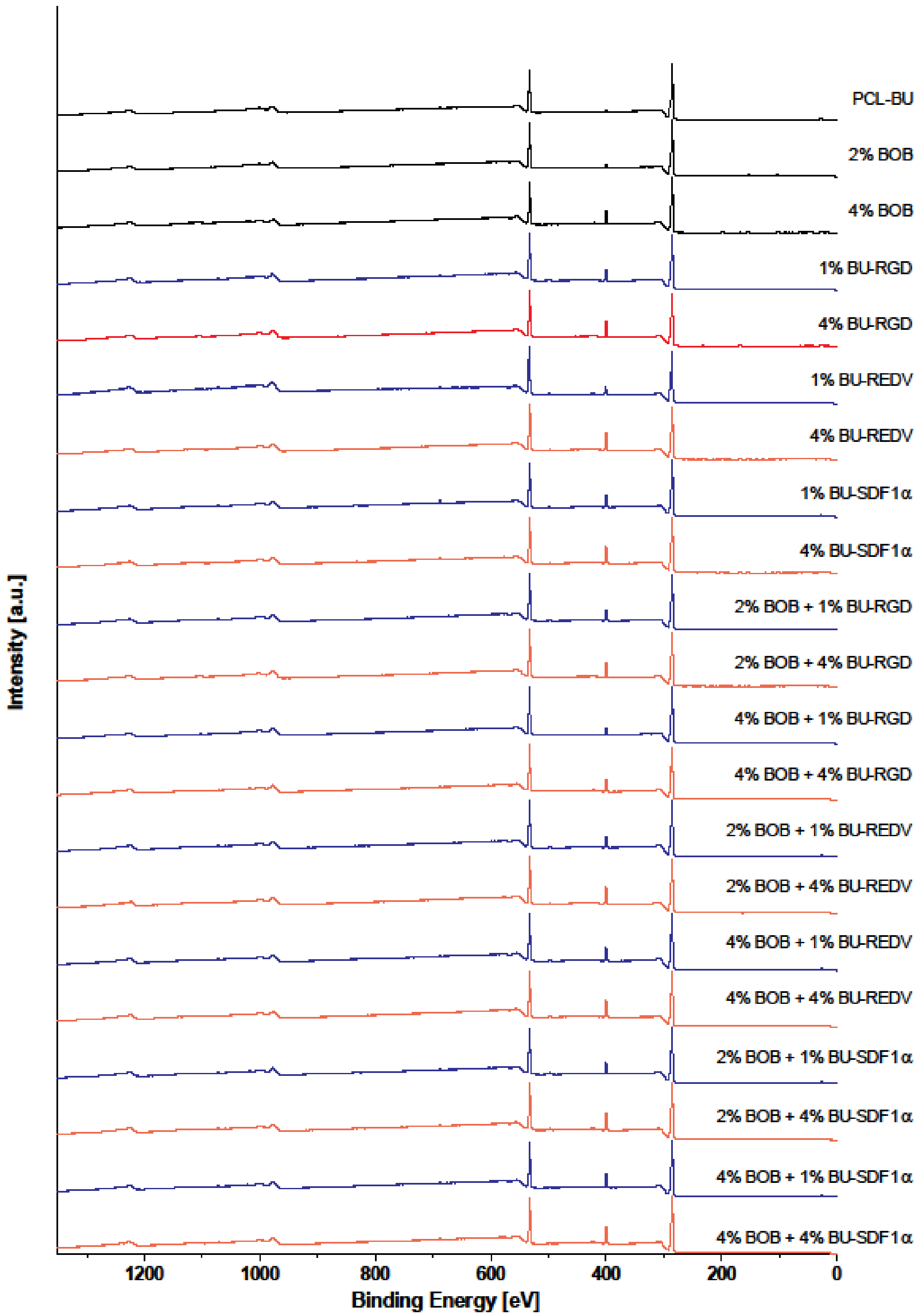

FIGURE S2 Normalized XPS survey spectra of solution-cast films of PCL-BU and combinations of BOB and bisurea-peptide conjugates.

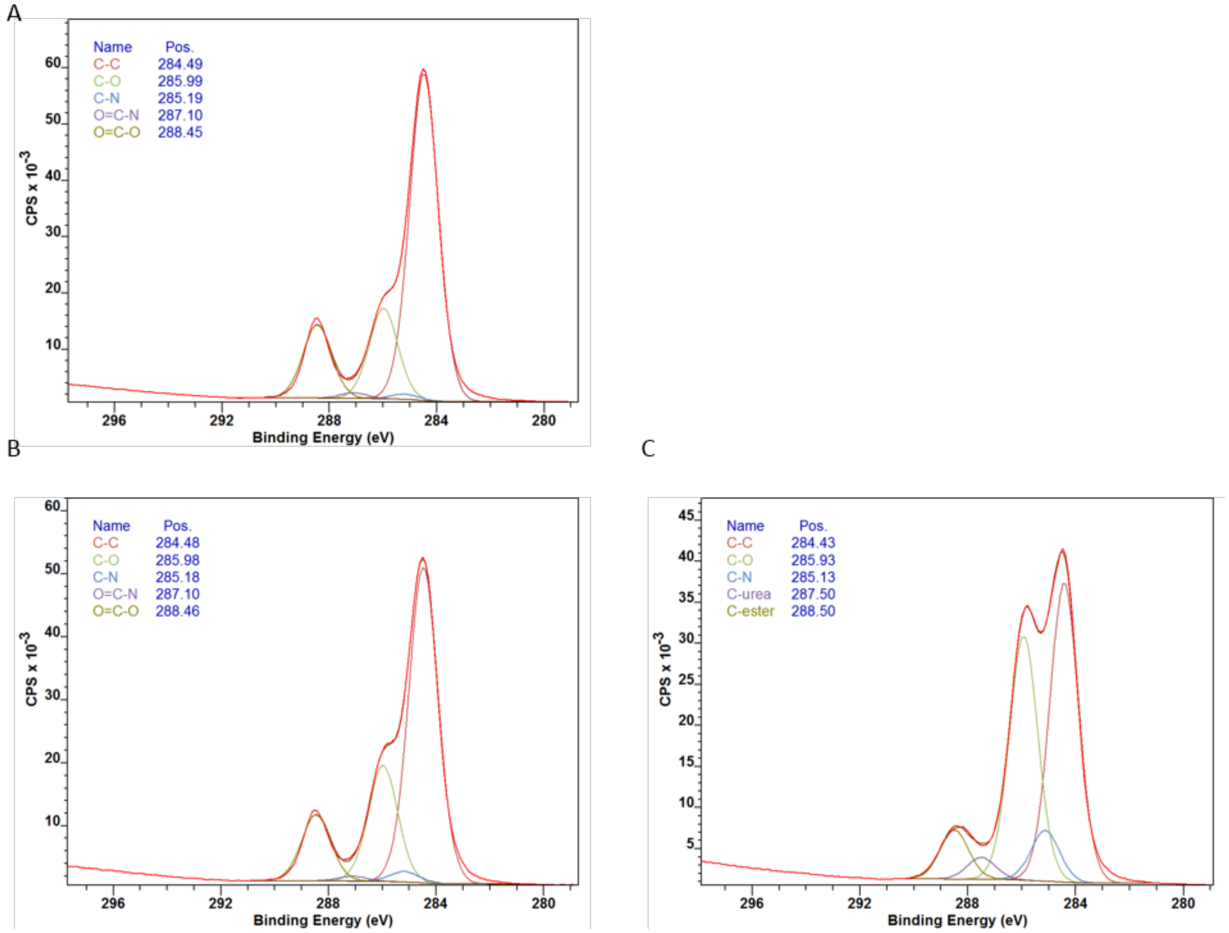

FIGURE S3 XPS narrow scans of carbon region for A) pristine PCL-BU B) 2% BOB and C) 4% BOB.

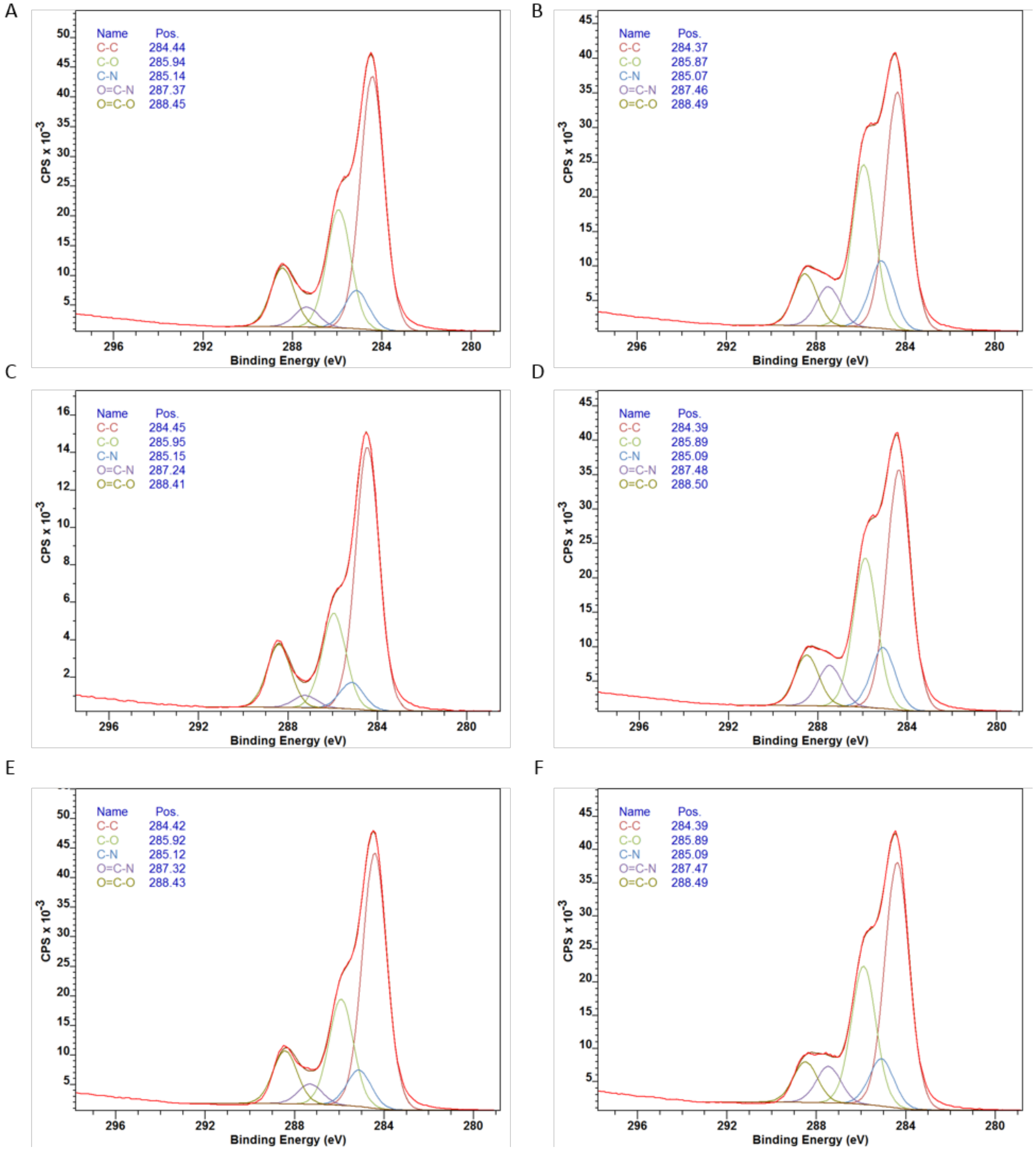

FIGURE S4 XPS narrow scans of carbon region for A) 1% BU-RGD, B) 4% BU-RGD, C) 1% BU-REDV, D) 4% BU-REDV, E) 1% BU-SDF1α, and F) 4% BU-SDF1α.

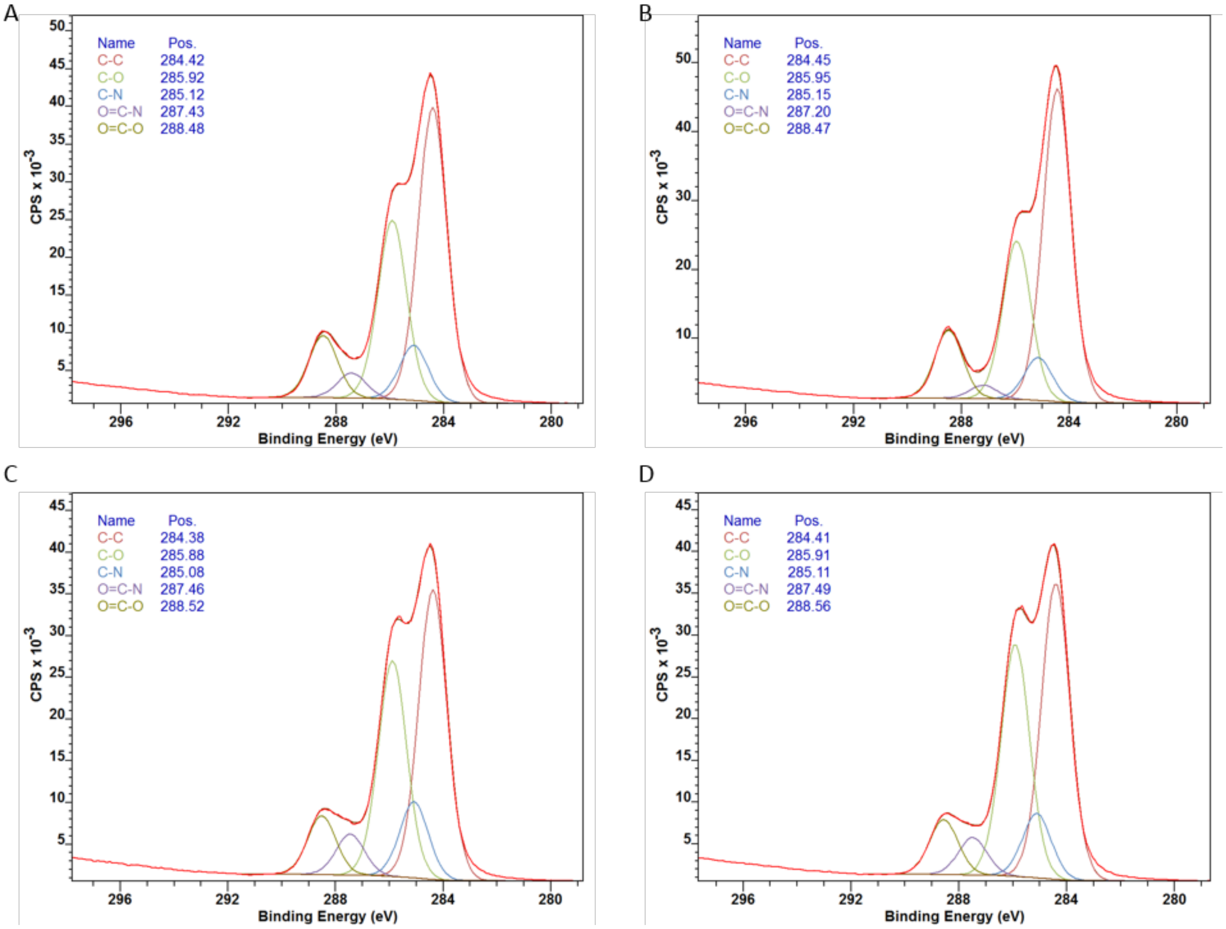

FIGURE S6 XPS narrow scans of carbon region for A) 2% BOB + 1% BU-RGD, B) 4% BOB + 1% BU-RGD, C) 2% BOB + 4% BU-RGD, and D) 4% BOB + 4% BU-RGD.

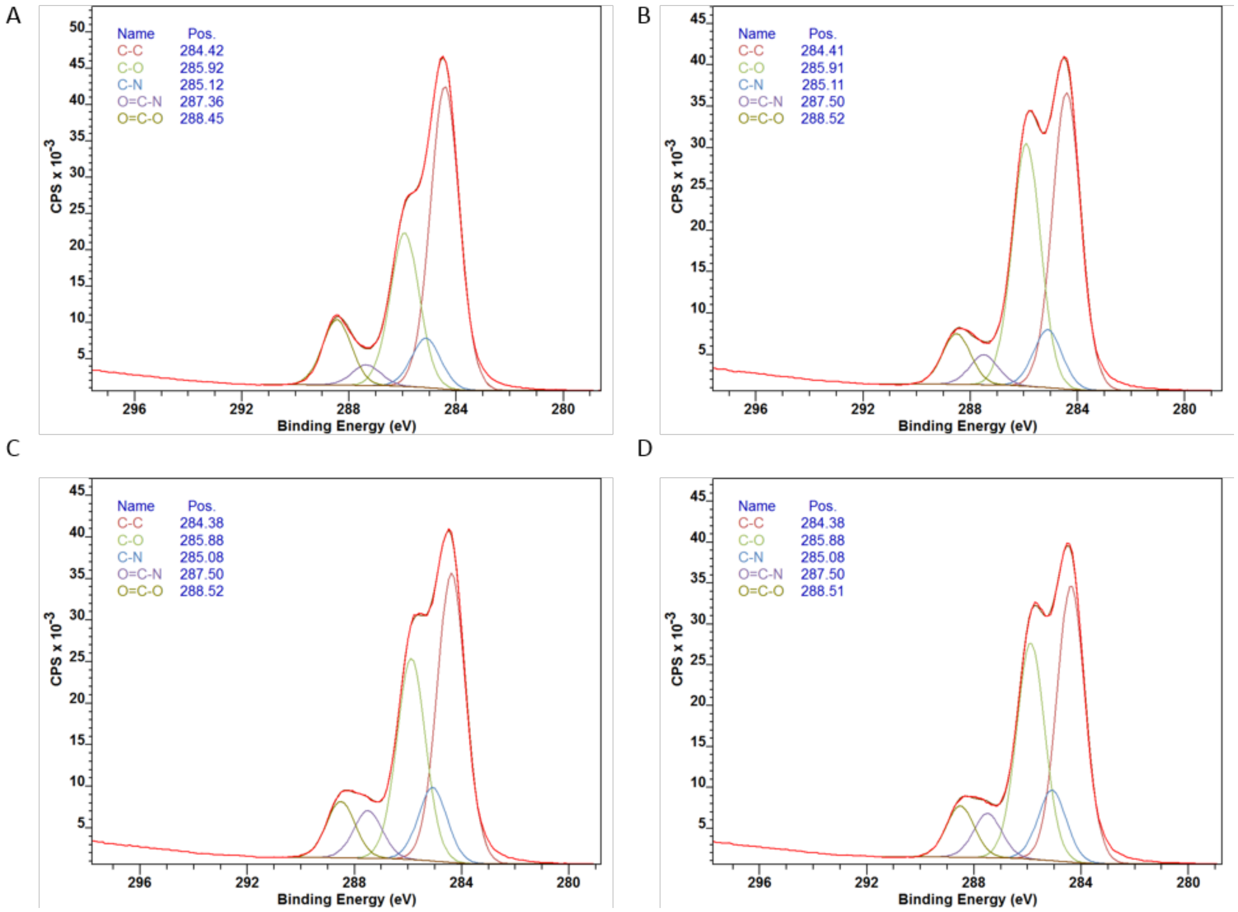

FIGURE S7 XPS narrow scans of carbon region for A) 2% BOB + 1% BU-REDV, B) 4% BOB + 1% BU-REDV C), 2% BOB + 4% BU-REDV, and D) 4% BOB + 4% BU-REDV.

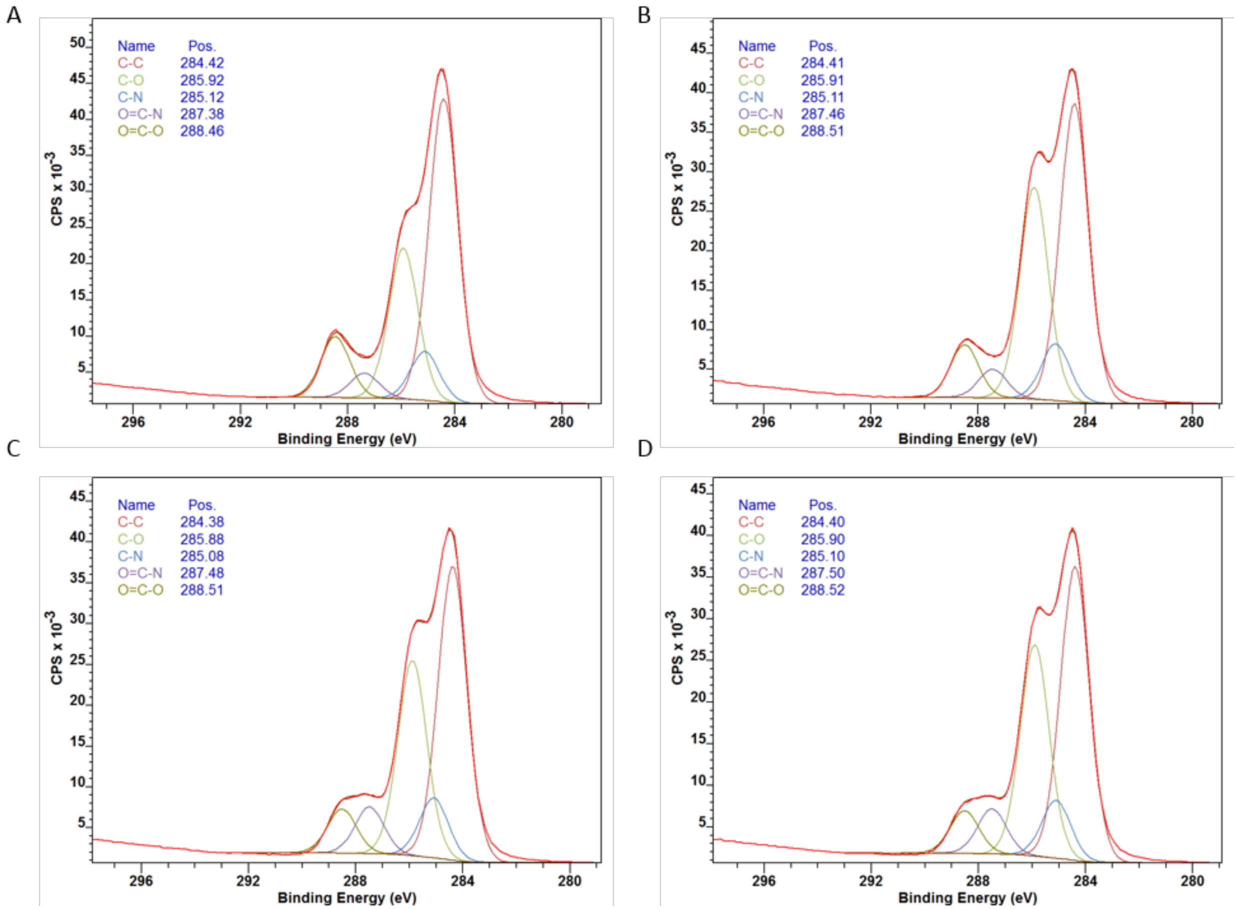

FIGURE S8 XPS narrow scans of carbon region for A) 2% BOB + 1% BU-SFD1α, B) 4% BOB + 1% BU- SFD1α C), 2% BOB + 4% BU- SFD1α, and D) 4% BOB + 4% BU- SFD1α.

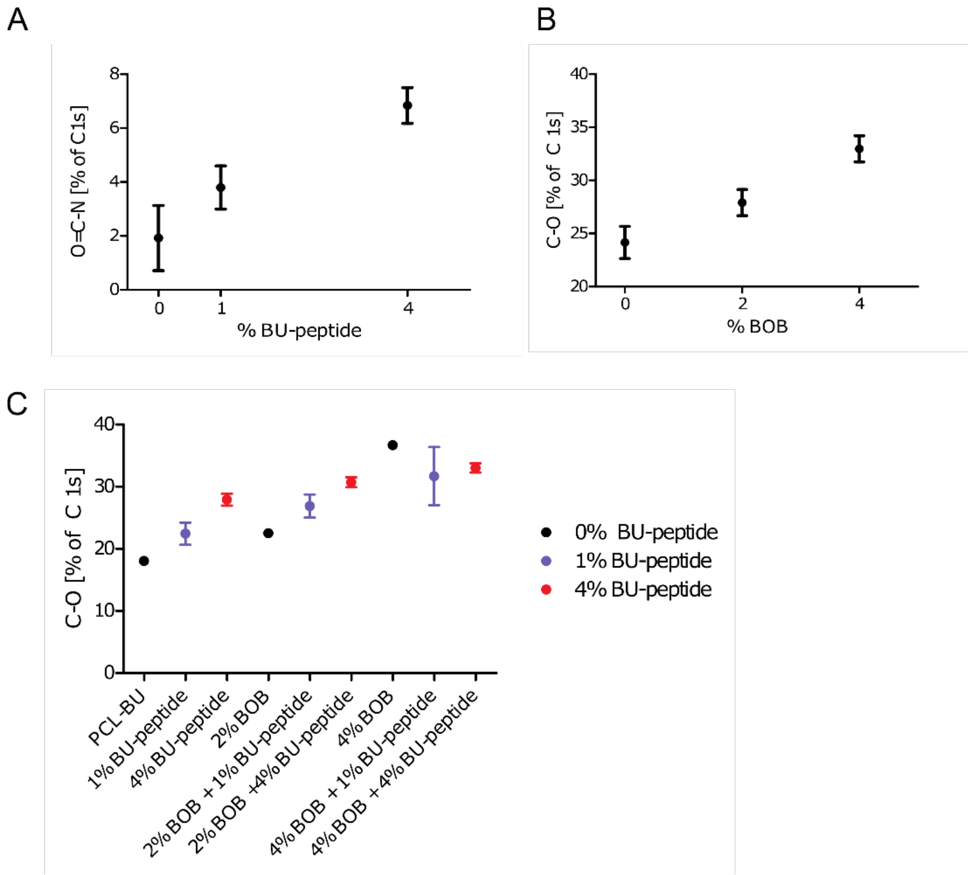

FIGURE S9 A) O=C-N component of Carbon 1s spectrum as a function of BU-peptide concentration. B) C-O component of C 1s spectrum as a function of BOB concentration, also in combination with the BU-peptide additives. C) C-O component of C 1s spectrum related to the concentration of both the antifouling and BU-peptide additives, for which the absolute amount of oligo(ethylene glycol) units increases incrementally. Data is represented as mean ± SD.

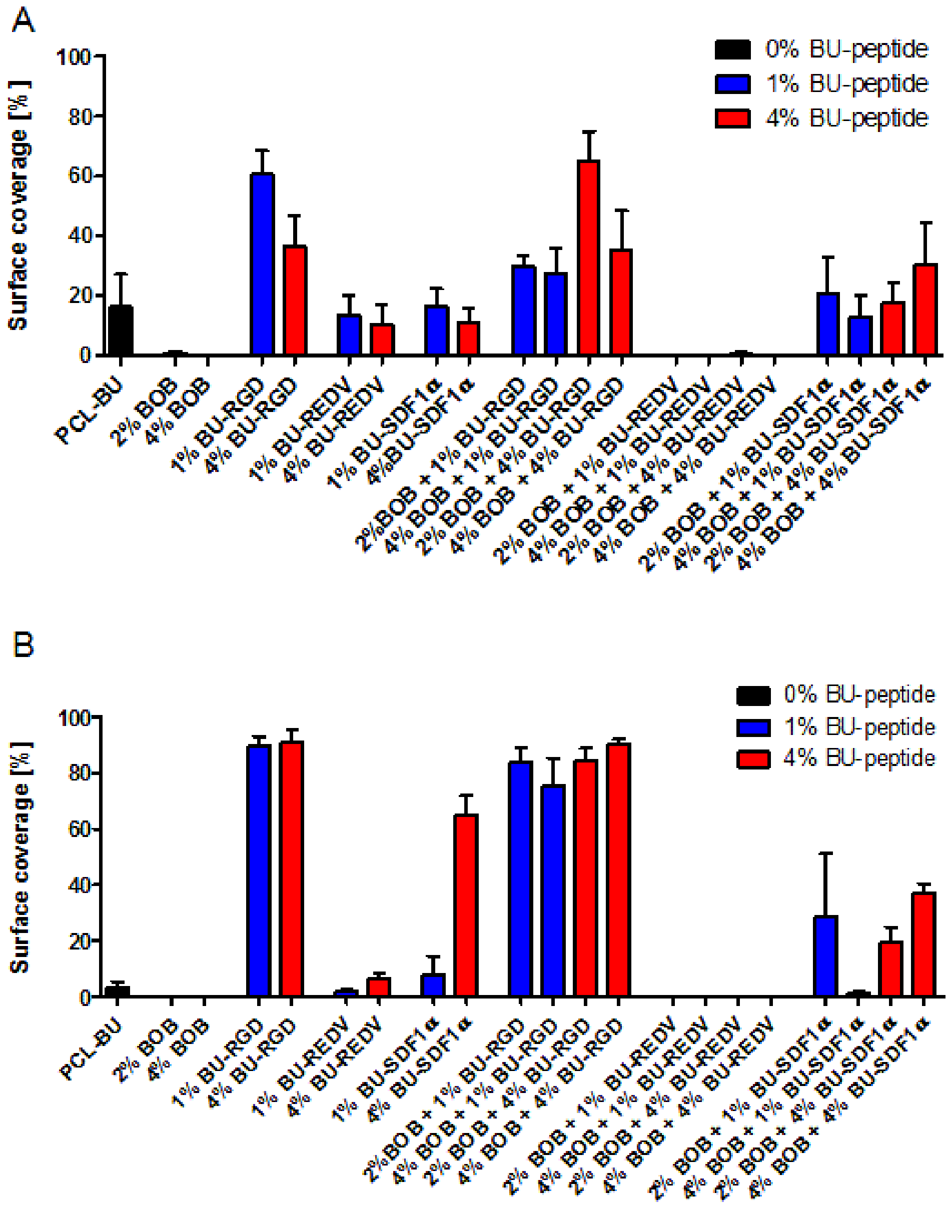

FIGURE S10 Surface coverage for Endothelial Cells (A) and Smooth Muscle Cells (B) on solution-cast films of the co-assembled BU-peptides and BOB in PCL-BU. Data is represented as mean ± SD.

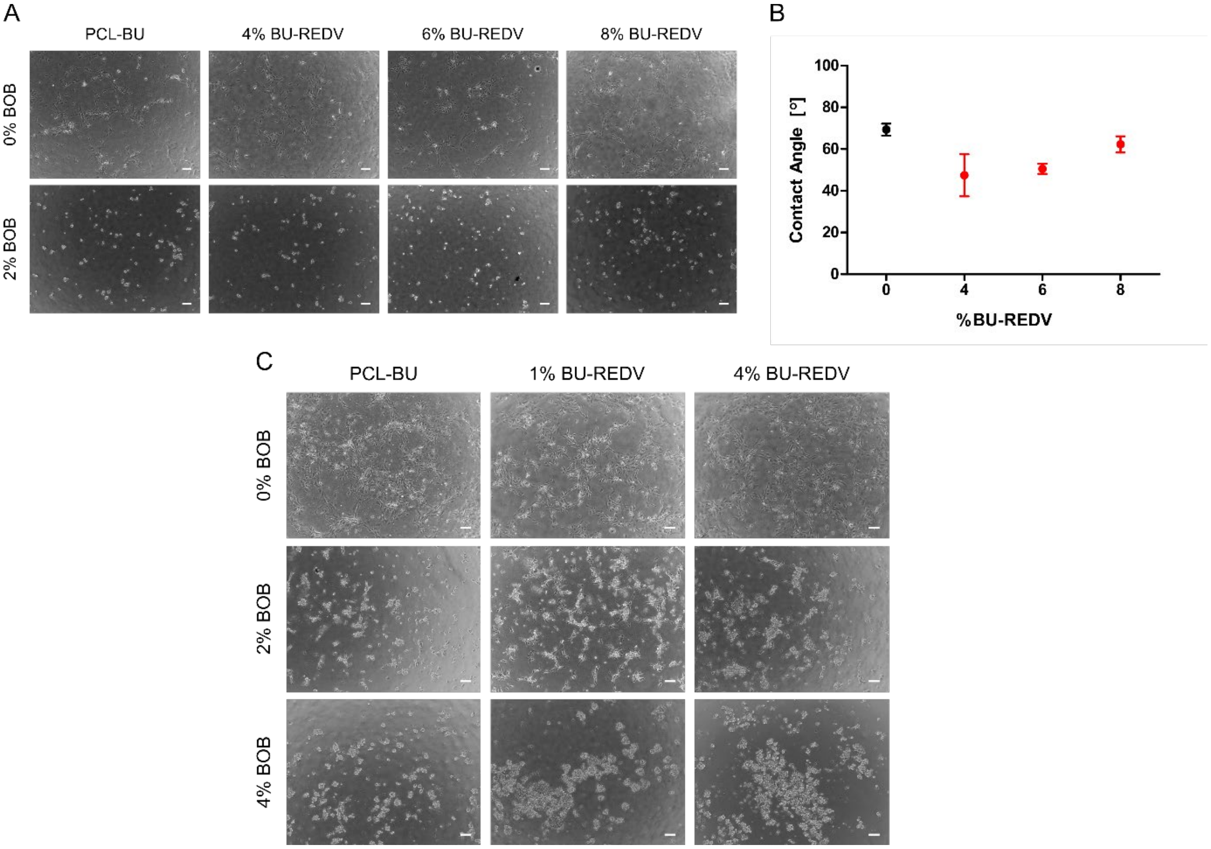

FIGURE S11 A) Phase contrast micrographs of human umbilical vein endothelial cells cultured for 24 hours on solution-cast films of PCL-BU functionalized with mixtures of 2 mol% BU-OEG-BU and increased BU-REDV concentrations. Scale bars need to be included. B) Water contact angles on solution-cast films of PCL-BU with 4, 6, and 8 mol% BU-REDV. Data is represented as mean ± SD. C) Phase contrast micrographs of human Aortic Endothelial Cells cultured for 24 hours on solution-cast films of PCL-BU functionalized with mixtures of BU-OEG-BU and BU-REDV. Scale bars represent 100 µm.

References

1. De Feijter, I., Goor, O. J. G. M., Hendrikse, S. I. S., Comellas-Aragonès, M., Söntjens, S. H. M., Zaccaria, S., Fransen, P. P. K. H., Peeters, J. W., Milroy, L. G., Dankers, P. Y. W. *Synlett*, **2015**, *26*, 2707.
